# Semaphorin7A regulates neuroglial plasticity in the adult hypothalamic median eminence

**DOI:** 10.1038/ncomms7385

**Published:** 2015-02-27

**Authors:** Jyoti Parkash, Andrea Messina, Fanny Langlet, Irene Cimino, Anne Loyens, Danièle Mazur, Sarah Gallet, Eglantine Balland, Samuel A. Malone, François Pralong, Gabriella Cagnoni, Roberta Schellino, Silvia De Marchis, Massimiliano Mazzone, R. Jeroen Pasterkamp, Luca Tamagnone, Vincent Prevot, Paolo Giacobini

**Affiliations:** 1 Inserm, Laboratory of Development and Plasticity of the Neuroendocrine Brain, Jean-Pierre Aubert Research Centre, U1172, 59045 Lille, France; 2 University of Lille, School of Medicine and Institut de Medecine Predictive et de Recherche Therapeutique (IMPRT-IFR114), 59045 Lille, France; 3 Service of Endocrinology, Diabetology and Metabolism, Faculty of Biology and Medicine, University Hospital, 1011 Lausanne, Switzerland; 4 Candiolo Cancer Institute—FPO, IRCCS, 10060 Candiolo, Italy; 5 Department of Oncology, University of Torino, 10060 Candiolo, Italy; 6 Dipartimento di Scienze della Vita e Biologia dei Sistemi, University of Torino, 10100 Torino, Italy; 7 Laboratory of Molecular Oncology and Angiogenesis, Vesalius Research Center, VIB, B-3000 Leuven, Belgium; 8 Laboratory of Molecular Oncology and Angiogenesis, Vesalius Research Center, Department of Oncology, KU Leuven, B-3000 Leuven, Belgium; 9 Department of Translational Neuroscience, Brain Center Rudolf Magnus, University Medical Center Utrecht, 3584 CG11 Utrecht, The Netherlands

## Abstract

Reproductive competence in mammals depends on the projection of gonadotropin-releasing hormone (GnRH) neurons to the hypothalamic median eminence (ME) and the timely release of GnRH into the hypothalamic–pituitary–gonadal axis. In adult rodents, GnRH neurons and the specialized glial cells named tanycytes periodically undergo cytoskeletal plasticity. However, the mechanisms that regulate this plasticity are still largely unknown. We demonstrate that Semaphorin7A, expressed by tanycytes, plays a dual role, inducing the retraction of GnRH terminals and promoting their ensheathment by tanycytic end feet via the receptors PlexinC1 and Itgb1, respectively. Moreover, Semaphorin7A expression is regulated during the oestrous cycle by the fluctuating levels of gonadal steroids. Genetic invalidation of Semaphorin7A receptors in mice induces neuronal and glial rearrangements in the ME and abolishes normal oestrous cyclicity and fertility. These results show a role for Semaphorin7A signalling in mediating periodic neuroglial remodelling in the adult ME during the ovarian cycle.

## Abstract (web summary)

Reproduction in mammals is dependent on the function of specific neurons that secrete gonadotropin-releasing hormone (GnRH) and project their axons to the median eminence (ME) of the hypothalamus. Here the authors show that Semaphorin7A signaling plays a role in mediating the plasticity of GnRH axon terminals and tanycytes in the ME.

## Graphical abstract

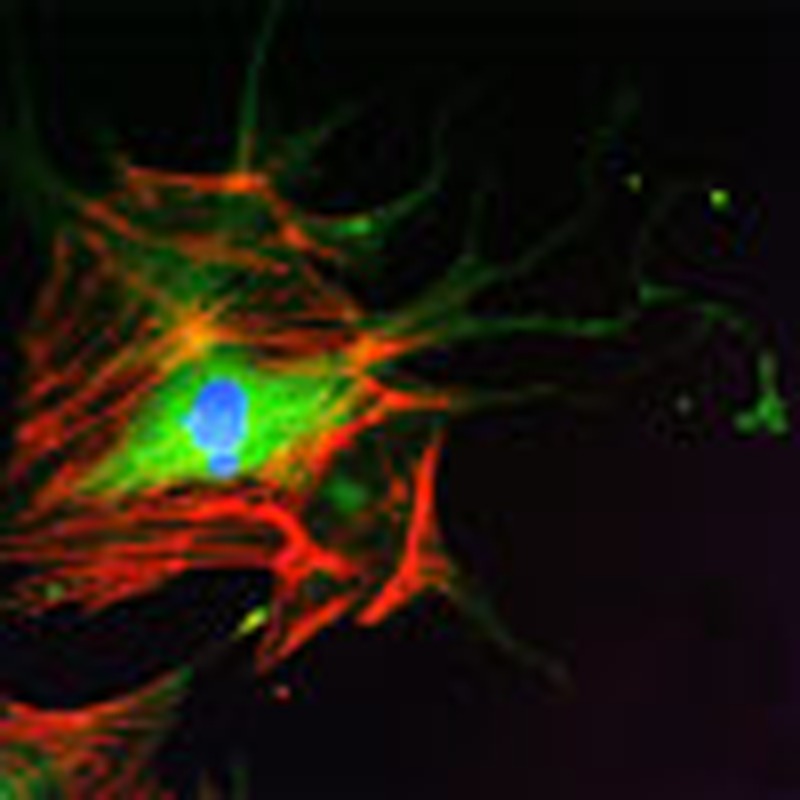

Reproduction in mammals is dependent on the function of specific neurons that secrete gonadotropin-releasing hormone (GnRH). These neurons project their axons to the median eminence (ME) of the hypothalamus, which serves as an interface between the neural and peripheral endocrine systems. Here, GnRH is released into the pituitary portal blood vessels for delivery to the anterior pituitary, to elicit the secretion of luteinizing hormone (LH) and follicle-stimulating hormone^1^. Alterations in the development of this system or in the secretion of GnRH are associated with the reduction or failure of sexual competence^2^.

It is increasingly recognized that in adult vertebrates, the GnRH neuroendocrine system displays striking structural and functional plasticity that is correlated with changes in the animal’s physiological state^3^. Remarkably, both GnRH neurons and the multiple neuronal networks involved in the control of GnRH secretion are subject to direct modulation by peripheral gonadal steroids^4^,^5^,^6^,^7^. During the ovarian cycle, under conditions of low gonadotropin output, GnRH-secreting axon terminals are distant from the pericapillary space of the ME, thus impairing the access of the neurohormone to the pituitary portal circulation^8^. There is now a growing body of evidence indicating that cell–cell interactions involving non-neuronal cells such as vascular endothelial cells, astrocytes and specialized ependymoglial cells named tanycytes, which ensheathe the terminals of GnRH neurons, might be of critical importance in the regulation of GnRH secretion^9^,^10^,^11^,^12^. However, the molecular mechanisms underlying this plasticity remain largely unknown.

Semaphorins are a family of soluble and membrane-bound proteins first identified as potent chemorepulsive axon guidance cues during development, where they play an essential role in neural-network formation^13^,^14^. For example, we have recently shown that Semaphorin7A (Sema7A) is essential for the development of the GnRH neuronal system, and that loss of Sema7A signalling during early development alters GnRH neuron migration, resulting in significantly reduced numbers of these neurons in the adult brain as well as in reduced gonadal size and subfertility^15^,^16^. Intriguingly, these molecules are also constitutively expressed in the postnatal brain and could thus regulate neuronal plasticity and nervous system physiology in adulthood^14^,^17^. We therefore asked whether Sema7A and its two well-characterized receptors PlexinC1 and β1-integrin (Itgb1) contribute to the periodic remodelling of the GnRH system in adulthood.

Our results reveal a role for Sema7A signalling in mediating the plasticity of GnRH neurons and tanycytes in the ME of the adult hypothalamus via PlexinC1 and Itgb1, respectively. In particular, our findings show that tanycytes, which insulate the pericapillary space of pituitary portal vessels from GnRH nerve terminals, express Sema7A, and that this expression is dynamically regulated during the oestrous cycle by fluctuating gonadal steroid levels. We also demonstrate that Sema7A induces rapid structural changes in the ME, impairing the direct access of GnRH axons to the portal vasculature, and that the inhibition of Rap1 is essential for the Sema7A-mediated collapse of GnRH neuronal growth cones in a PlexinC1-dependent manner. Moreover, we show that tanycytes play an active role in mammalian reproduction via Sema7A/Itgb1 signalling.

## Results

### Sema7A expression in the ME changes during the oestrous cycle

As Sema7A is expressed postnatally in the ME^18^, where GnRH terminals secrete their neurohormone, we investigated whether this signalling molecule could play a role in the correct functioning of the GnRH system during adulthood.

**Figure 1:**
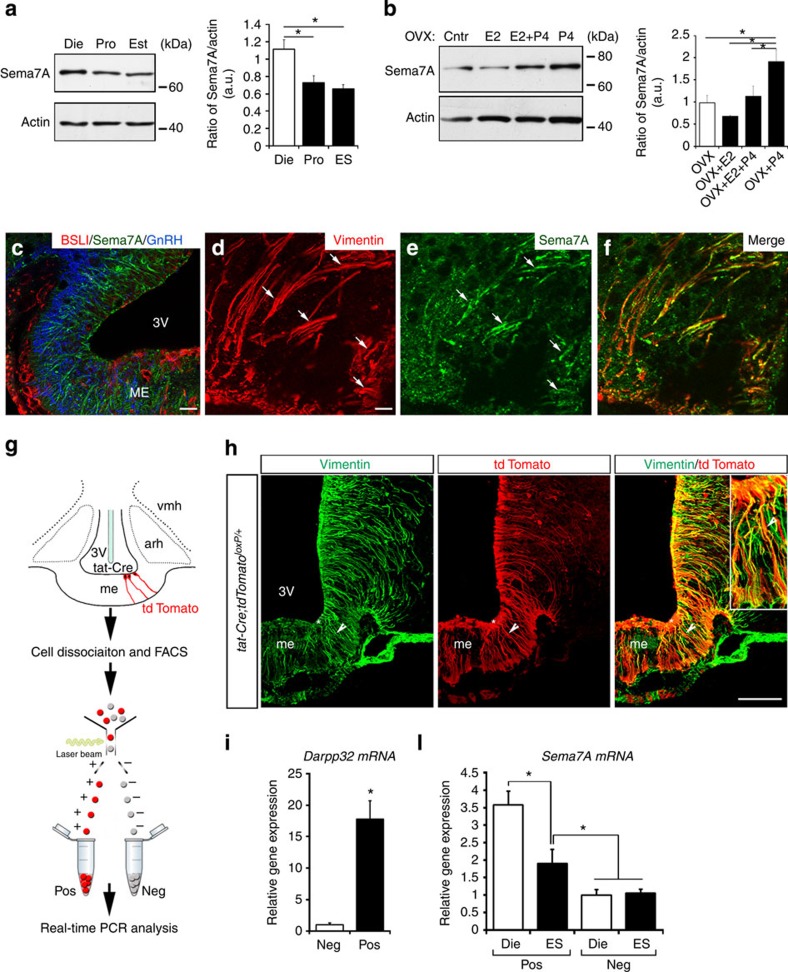
Sema7A expression in ME tanycytes varies as a function of the oestrous cycle. (**a**) Western blotting for Sema7A in the ME during the oestrous cycle. Bar graph illustrates the mean ratio (±s.e.m.) of the signal intensity obtained for Sema7A to that of total actin (*n*=4 independent experiments per oestrous cycle phase; 3- to 4-month-old females rats, Kruskal–Wallis test. *P<0.05, values are normalized to the Diestrus group). (**b**) Western blot analysis for Sema7A and actin in the ME of control OVX rats and those treated with saline, 17β-estradiol 3-benzoate (OVX+E2), progesterone (OVX+P4) and 17β-estradiol 3-benzoate+progesterone (OVX+E2+P4). *N*=4; 3- to 4-month-old females rats, Kruskal–Wallis test *P<0.05, values are normalized to the OVX group. (**c**–**f**) Immunofluorescence for Sema7A, GnRH, BSLI and vimentin in coronal sections of the rat ME (*n*=4 brains for each experiment, 3- to 4-month-old females rats. Experiments were repeated five times). (**c**) Simultaneous triple-labelling experiments for Sema7A (green), GnRH (blue) and the endothelial-cell marker BSLI (red). (**d**–**f**) Confocal images of double immunofluorescence for Sema7A (green) and vimentin (red). Tanycytic processes (**d**, arrows) exhibit Sema7A immunoreactivity (**c**, arrows, **f**). 3 V, third ventricle. (**g**) Tanycyte isolation by FACS and real-time PCR analysis in Tomato-positive (pos; tanycytes) and -negative cells (neg) in *tdTomato*^*loxP/+*^ mice infused i.c.v. with Tat-cre. (**h**) Tat-cre injection into the third ventricle results in Cre-Lox recombination (red) exclusively in tanycytes (vimentin immunoreactive, green), whose cell bodies line the wall of the third ventricle (asterisks). (**i**) Real-time PCR analysis of *DARPP32* (*n*=5 *Tat-cre; tdTomato*^*loxP/+*^) mRNA in tanycytes isolated from *Tat-cre;tdTomato*^*loxP/+*^ mice, normalized to values in Tomato-negative cells. *P<0.01, Wilcoxon–Mann–Whitney test. (**l**) Real-time PCR analysis of *Sema7A* mRNA in tanycytes isolated from *Tat-cre;tdTomato*^*loxP/+*^ mice, normalized to values in Tomato-negative cells isolated from *Tat-cre;tdTomato*^*loxP/+*^ mice at diestrus and estrus (*n*=4 Diestrus *Tat-cre; tdTomato*^*loxP/+*^ versus *n*=4 Estrus *Tat-cre; tdTomato*^*loxP/+*^). *P<0.05, Kruskal–Wallis test. Values shown are means±s.e.m. Scale bars, (**c**) 40 μm, (**d**–**f**) 80 μm, (**h**) 100 μm.

GnRH nerve terminals are located in close proximity to the pericapillary space of pituitary portal blood vessels in the ME during proestrus, in preparation for neurohormone release, but they retract during diestrus^19^ as well as after gonadectomy^20^, when GnRH secretion is low. To investigate whether this periodic growth and retraction could be related to Sema7A levels, we used western blotting to examine the expression of Sema7A in the ME of female rats in diestrus II, in proestrus at the onset of the preovulatory stage and during estrus. We found that the expression of Sema7A was significantly increased on the day of diestrus as compared with that of proestrus and estrus (Fig. 1a). Quantification of Sema7A levels showed maximal levels on the afternoon of diestrus II and thereafter progressively declining expression during proestrus and estrus (diestrus versus proestrus versus estrus, all at 1600, h).

To confirm these changes in Sema7A levels and confirm that they are sex-steroid-dependent, we ovariectomized (OVX) cycling adult female rats and subsequently treated these animals with subcutaneous (s.c.) injections of sesame oil alone, or containing 17β-estradiol 3-benzoate (E2), progesterone (P4) or E2+P4. As shown in Fig. 1b, P4 induced a significant increase of Sema7A expression in the ME of OVX rats as compared with the other groups (Fig. 1b).

### Sema7A expression varies in response to ovarian steroids

The ME is one of eight circumventricular organs—regions surrounding the cerebral ventricles—in the central nervous system, in which the blood–brain barrier is modified to allow the release of neurohormones produced by neuroendocrine cells from terminals into the pituitary portal blood vessels for delivery to the anterior pituitary^21^. The ME contains nerve terminals, tanycytes, astrocytes and fenestrated endothelial cells. We next determined the identity of the cell type(s) expressing Sema7A in the ME and the factors regulating this expression.

We determined the expression pattern and cellular source of Sema7A in the ME by fluorescent labelling experiments using BSLI (Bandeiraea simplicifolia lectin I), which labels endothelial cells and antibodies directed against Sema7A, vimentin and GnRH, in the adult rat hypothalamus (Fig. 1c–f). Although no Sema7A immunoreactivity was associated with endothelial cells or GnRH terminals (Fig. 1c), we detected robust Sema7A expression in tanycytes, identified by immunolabelling for vimentin^22^ (Fig. 1d–f). No Sema7A protein was detected in the ME of *Sema7A*^*−/−*^ mutant mice (Supplementary Fig. 1a-d).

To determine whether the differences in Sema7A protein levels in ME demonstrated above were due to modulation of tanycytes’ expression as a function of fluctuating gonadal steroids, we next examined Sema7A transcript expression in tanycytes by stereotaxically injecting the Tat-cre fusion protein^23^ into the third ventricle of *tdTomato*^*loxP/+*^ reporter female mice (Fig. 1g). We have recently shown that this method selectively achieves reporter fluorescent protein expression in tanycytes, as opposed to neurons, astrocytes or endothelial cells^24^. Cre recombination occurred in virtually all vimentin-immunoreactive cells of the ME (Fig. 1h).

We then isolated Tomato-positive cells from explants containing both the ME and the mediobasal hypothalamus by fluorescence-activated cell sorting (FACS) and used quantitative reverse transcriptase–PCR (RT–PCR) to verify the expression of the tanycytic marker DARPP32 (ref. 25) (Fig. 1i). Sorted Tomato-positive cells abundantly expressed *DARPP32*, which was barely detectable in Tomato-negative cells^24^ (Fig. 1i). We therefore isolated Tomato-positive and -negative cells from female mice in diestrus and estrus, and performed quantitative RT–PCR profiling for *Sema7A* (Fig. 1l).

It has been reported previously that Sema7A is expressed by hypothalamic neurons as well as in the ME^18^. Consistently, Sema7A was expressed in both Tomato-positive and -negative cells. Only tanycytic (Tomato-pos) Sema7A expression varies as a function of the hormonal status of the animal (Fig. 1l), whereas in Tomato-negative cells, Sema7A expression does not change between diestrus and estrus.

We next determined whether tanycytes could respond directly to changes in circulating gonadal steroid levels by verifying the expression of oestrogen (ERα and ERβ) and progesterone receptors (PR-A/B), using RT–PCR on Tomato-positive and -negative cells isolated as described above (Supplementary Fig. 2a). Tomato-positive cells expressed *ERα, ERβ* and *PR-A/B* messenger RNAs, confirming that the cyclic variation in Sema7A expression by tanycytes could indeed be directly controlled by gonadal steroids.

**Figure 2:**
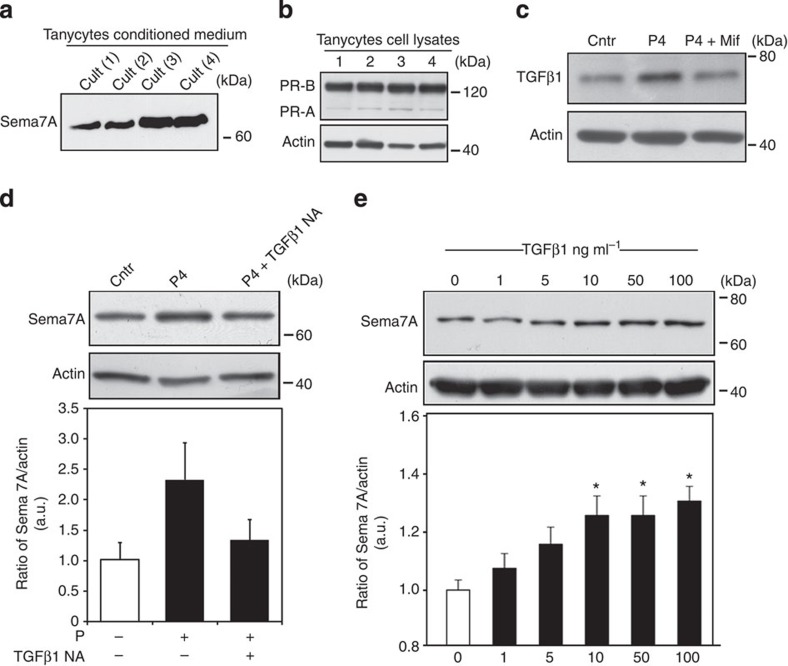
Tanycytic Sema7A expression is progesterone and TGF-β1 dependent. (**a**) Western blot analysis of Sema7A-secreted protein in the conditioned media (CM) of four representative tanycytic cultures 48 h after plating. (**b**) Representative western blotting showing the expression of progesterone receptors, PR-A and PR-B, in primary tanycytes cell lysates (*n*=4). Primary tanycytes were treated with progesterone (P) with or without its antagonist Mifepristone (Mif), and TGF-β1 (**c**) and Sema7A (**d**) expression were evaluated by immunoblotting on total cell lysates (*n*=3 independent experiments for each treatment condition). (**e**) Representative immunoblot depicting a TGF-β1 dose–response experiment (6 h of treatment) on Sema7A protein levels in primary tanycytes. Bar graphs illustrate the mean ratio of the signal intensity obtained for Sema7A to that for total actin (*n*=3 independent experiments per treatment; Kruskal–Wallis test *P<0.05; values are normalized to the control groups).

Earlier studies have demonstrated that Sema7A expression is regulated by transforming growth factor (TGF)-β1 in non-neuronal tissues^26^. As TGF-β1 is expressed in the ME^27^, including in tanycytes (Supplementary Fig. 2b), and because its expression is known to fluctuate in the hypothalamus of adult female rats during the oestrous cycle^28^, we next studied whether progesterone regulates Sema7A expression in these cells by modulating TGF-β1 expression. We generated primary cultures of tanycytes from the rat ME (Fig. 2a–e) that we had previously extensively characterized^29^,^30^, as FACS-sorted tanycytes do not provide enough protein for western blotting.

Sema7A can act either as a membrane-bound molecule or as a soluble factor following proteolytic cleavage^13^; we thus verified that tanycytes express (Fig. 2d,e) and secrete Sema7A into the culture medium (Fig. 2a).

To explore the role of P4, we used western blotting to confirm the presence of progesterone receptors (PR-A and -B) in these primary tanycyte cultures and analyse the expression TGF-β1 and Sema7A proteins in total cell lysates (Fig. 2b). Coherently with the *in vivo* data, primary tanycytes exposed to P4 (100 nM for 6 h) expressed higher levels of TGF-β1 and Sema7A as compared with untreated controls (Fig. 2c,d), an effect that was specific, as shown by the lack of a response following the administration of P4 together with its antagonist, mifepristone (Fig. 2c). Moreover, the effect of P4 on Sema7A expression was modulated by TGF-β1, as it was prevented when tanycytes were treated with a TGF-β1-neutralizing antibody in addition to progesterone (Fig. 2d). Treatment of primary tanycyte cultures for 6 h with TGF-β1 promoted Sema7A protein expression in a dose-dependent manner (with plateau activation at 10 ng ml^−1^), thus confirming that TGF-β1 is upstream Sema7A induction (Fig. 2e).

These findings show that increased Sema7A levels in diestrus are controlled by progesterone, via the local cyclic release of TGF-β1-regulating hypothalamic tanycytes in an autocrine/paracrine manner.

### Sema7A induces morphological plasticity in the ME

**Figure 3:**
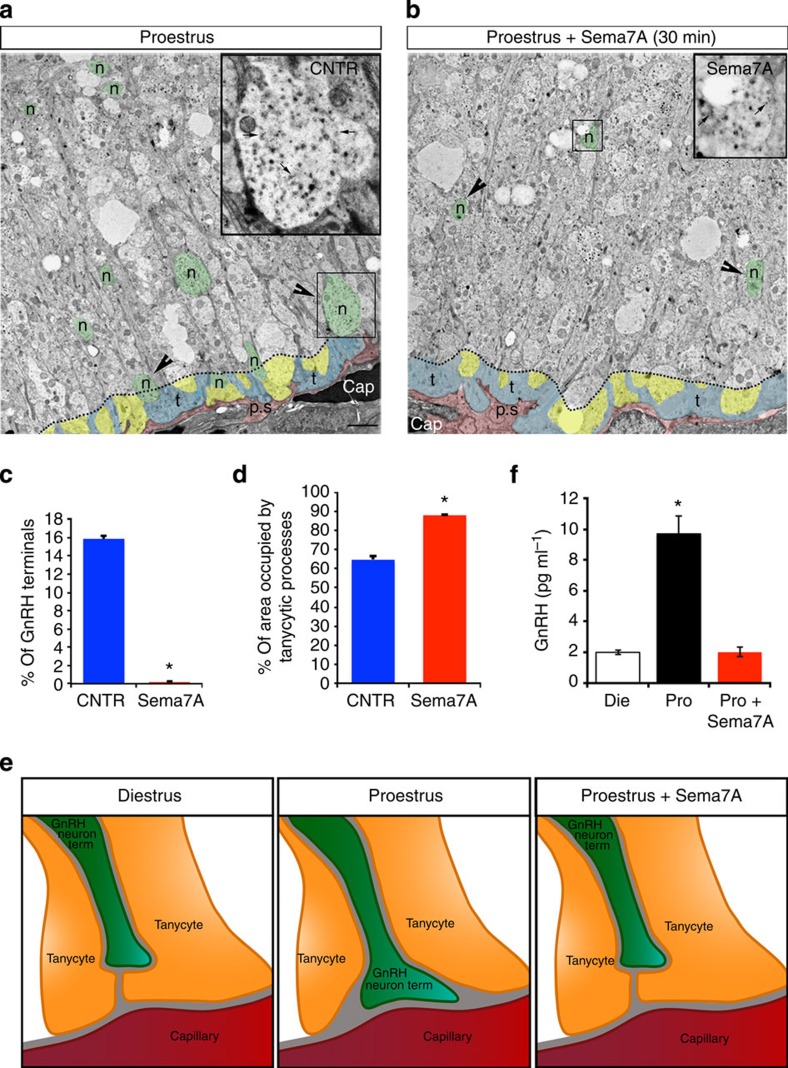
Sema7A induces morphological plasticity in the ME of the adult female brain. (**a**,**b**) Representative electron micrographs of GnRH-immunoreactive axon terminals (immunogold) from hypothalamic explants containing the ME of female rat in proestrus, incubated in the absence (**a**) or the presence (**b**) of Sema7A (1 μg ml). GnRH axonal endings are highlighted in green, other neuroendocrine nerve terminals in yellow and the pericapillary space (p.s.) in pink. (**c**) Quantitative analysis of the percentage of GnRH nerve terminals located <1 μm from the pericapillary space in the external zone of the ME, in explants from rats (3–4 months old mice) in proestrus treated or not with Sema7A (*n*=4 animals per condition; 100–150 GnRH-immunoreactive axon terminals were considered per explant; Wilcoxon–Mann–Whitney test, *P<0.05). (**d**) Quantitative analysis of the percentage of area occupied by tanycytes in the external zone of the ME at <2 μm from the pericapillary space, in explants from rats in proestrus treated or not with Sema7A (*P<0.05; Wilcoxon–Mann–Whitney test; *n*=5 in controls, *n*=3 Sema7A-treated mice). (**e**) The schematic highlights the morphological changes in GnRH terminals and tanycytic end-feet processes during the different phases of the ovulatory cycle. (**f**) Quantification of GnRH secretion from ME explants dissected from adult female rats, during diestrus (*n*=6) or proestrus (*n*=6), stimulated or not for 4 h with Sema7A (250 ng ml^−1^; *n*=6). Conditioned medium was collected for each culture condition and processed by GnRH ELISA assay. GnRH concentrations are represented as means±s.e.m. One-way ANOVA followed by Tukey’s *post-hoc* test, *P<0.01. Scale bars, (**a**,**b**) 2 μm.

We have previously shown that GnRH axon terminals, which are located in close proximity to the pericapillary space surrounding pituitary portal blood vessels in the ME during proestrus (the ME/GnRH nerve terminals morphology in estrus is identical to the one seen in diestrus), retract during the subsequent phases of the oestrous cycle^19^,^31^. To assess whether this retraction is regulated by Sema7A, we analysed hypothalamic explants containing the ME, maintained *ex vivo* in artificial cerebrospinal fluid^32^. Explants were treated with 1 μg ml^−1^ Sema7A for 30 min, fixed and processed for electron microscopy using 15 nm gold-particle labelling. As a function of the presence or absence of Sema7A during proestrus, GnRH nerve terminals underwent a robust transformation, with the distance between these terminals (green) and the pericapillary space (pink) increasing significantly in Sema7A-treated explants (Fig. 3a,b). A quantitative morphometric analysis showed that although the total number of GnRH nerve terminals at a distance of 10 μm from the parenchymatous basal lamina (which delineates the pericapillary space) did not vary significantly between treatments (*n*=4 animals per condition; 100–150 GnRH-immunoreactive axon terminals were considered per explant; Wilcoxon–Mann–Whitney test, *P*=0.42), their distribution was markedly changed (Fig. 3c). In fact, only a few GnRH-immunoreactive terminals located at <1 μm from the pericapillary space were detected in ME explants, dissected at proestrus and exposed to Sema7A, as compared with controls (Fig. 3a–c). The choice of 1-μm limit for this quantitative analysis was made based on previous studies of the spatial distribution of GnRH nerve terminals in the external zone of the ME, which demonstrated that marked structural changes only occur for the fraction of GnRH nerve terminals located at <1 μm from the pericapillary space (not at 2, 3 or 10 μm distance from the basal lamina)^33^.

### Bifunctional role of Sema7A in the hypothalamic ME

The axon terminals of GnRH neurons are engulfed by tanycyte processes, which periodically remodel under the influence of circulating gonadal steroids to further restrict the proximity of GnRH terminals to the pericapillary space of the ME^12^. We thus evaluated whether Sema7A treatment might influence these morphological rearrangements in tanycytes. Owing to their anatomical organization (tanycyte processes are always closely associated to the parenchymatous basal lamina), this second analysis was performed by analysing the area occupied by tanycytic end feet rather than their distance from the pericapillary space. For this purpose, the analysis was performed within a 2-μm limit from the basal lamina of the ME, where the morphological changes are more evident.

Following Sema7A stimulation, the area occupied by tanycytic end feet (blue) rose from 63% to 88%, and only a few scattered neuroendocrine nerve endings (yellow) were still present (Fig. 3d). These data show that Sema7A induces the retraction of GnRH nerve terminals and concomitantly promotes the expansion of tanycytic processes, potentially making the pericapillary space inaccessible to these nerve terminals (Fig. 3e).

We then generated organotypic cultures of ME explants dissected from adult female rats during diestrus II or proestrus, and challenged them with Sema7A (250 ng ml^−1^) for 2 h before using enzyme-linked immunosorbent assay (ELISA) to measure the amount of GnRH secreted into the medium (Fig. 3f). Under control conditions, the concentration of GnRH detected in our explant media remained low during diestrus II (Die) and rose during proestrus (Pro), as expected. Following Sema7A stimulation, the amount of GnRH secreted at proestrus was reduced to diestrus levels (Fig. 3f).

Taken together, our data demonstrate that Sema7A treatment of proestrus ME explants induces morphological changes of GnRH and tanycytic processes, as well as modification of GnRH secretion reminiscent of a diestrus state (Fig. 3e,f).

We next investigated the expression patterns of Sema7A receptors, Itgb1 and PlexinC1, in the ME, using immunofluorescence labelling and confocal microscopy (Supplementary Fig. 3a–l). We confirmed that GnRH-positive axon terminals located in the external zone of the ME co-expressed both Itgb1 (Supplementary Fig. 3a-d) and PlexinC1 (Supplementary Fig. 3e–h). In addition, many Itgb1-immunoreactive fibres were distributed throughout the internal zone and external zone of the ME, where axons of other populations of neuroendocrine neurons and tanycytic processes are located (Supplementary Fig. 3a,i). Double-labelling experiments for PlexinC1 and vimentin demonstrated that PlexinC1 is not expressed by ME tanycytes, and confirmed that PlexinC1 immunoreactivity is restricted to the external zone of the ME (Supplementary Fig. 3i-l). The same antibodies were previously used to show the differential expression of Itgb1 and PlexinC1 in GnRH neurons during embryonic development^15^. Moreover, we further tested the specificity of the PlexinC1 antibody on brain sections of transgenic mice lacking *PlexinC1* (Supplementary Fig. 1e–h).

### Sema7A signalling in the retraction of GnRH neurites

As biochemical studies of GnRH neurons are challenging because of their limited number (800 in mice and 1,000–2,000 in primates) and wide dispersal throughout the brain, we used GnV3 cells, one of 11 clones of GnRH-expressing cells obtained by the conditional immortalization of adult rat hypothalamic cell cultures^34^, to perform subsequent functional and biochemical experiments. Addition of doxycycline to the medium significantly stimulates GnV3 cell proliferation, whereas removal of this compound induces GnV3 cells to stop proliferating, differentiate and develop many of the features of mature adult GnRH neurons, including axon growth^34^,^35^. We first carried out western blot analyses to verify the expression of Sema7A receptors in this model of GnRH neurons (Supplementary Fig. 4a). Both PlexinC1 and Itgb1 were detected in GnV3 cells, confirming our observations in the whole brain and the pertinence of this model.

Sema7A stimulation of GnV3 cells activated ERK1/2 and increased the level of cofilin phosphorylation, which is known to inactivate cofilin by inhibiting its F-actin depolymerizing and severing activity^36^,^37^ (Supplementary Fig. 4a), and to be a pivotal signal transducer downstream of PlexinC1 (refs 38, 39).

**Figure 4:**
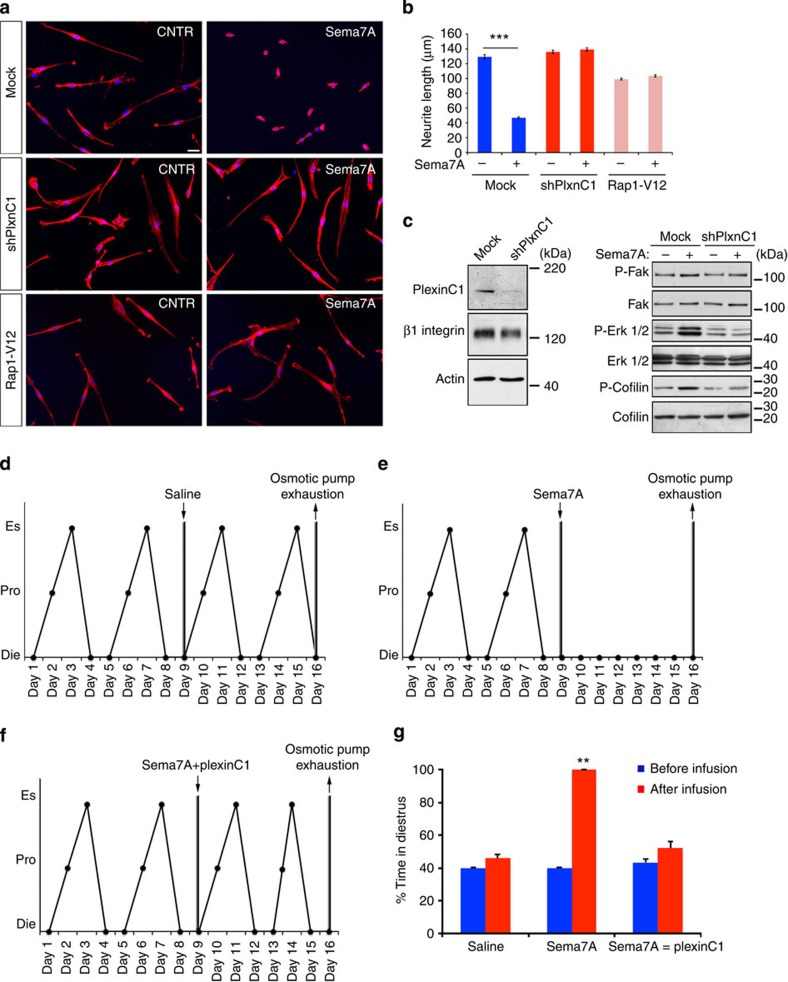
Sema7A induces the retraction of GnRH neurites through the PlexinC1 transduction pathway and Rap1 inactivation. (**a**) Representative images of semaphorin-induced neurite retraction in GnV3 neurons transfected with an empty vector (mock), a construct encoding a PlexinC1 short hairpin RNA (GnV3 shRNA PlexinC1) or a Rap1-V12 plasmid encoding a constitutively active form of Rap1. One day following Sema7A treatment (250 ng ml^−1^), cells were labelled for F-actin (red) to visualize cytoskeletal changes. (**b**) Quantitative analysis of neurite length in GnV3 cells under different treatment conditions (*n*=5 independent experiments, *n*=303 mock-transfected control cells, *n*=303 mock-transfected cells after Sema7A treatment, unpaired Student’s *t*-test, ***P<0.0001; *n*=267 shPlxnC1-treated cells, *n*=235 shPlxnC1+Sema7A-treated cells, unpaired Student’s *t*-test, *P*>0.05; *n*=131 Rap1-V12-transfected cells, *n*=115 Rap1-V12-transfected cells+Sema7A, unpaired Student’s *t*-test, *P*>0.05). (**c**) Immunoblotting for markers indicated using transfected GnV3 cells. (**d**–**f**) Saline, Sema7A or Sema7A+PlexinC1 was infused (0.2 μg μl^−1^, 0.5 μl h^−1^ for 7 days) by stereotaxic implantation of a 28-gauge infusion cannula connected to a subcutaneously implanted mini-osmotic pump in the ME of cycling female rats. Representative oestrous cycle profiles showing the disruption of oestrous cyclicity by the infusion of Sema7A but not of PBS into the ME. Infusion was started on day 9 (downward arrow) and ended 7 days later (upward arrow), when pump contents were exhausted. Die, Diestrus; Es, estrus; Pro, proestrus. (**g**) Quantitative analysis of alterations in ovarian cyclicity (percentage of time in diestrus) caused by PBS, Sema7A or Sema7A+soluble PlexinC1 infusion into the rat ME (*n*=6 animals per group, Kruskal–Wallis test, **P<0.005). Scale bar, (**a**) 10 μm.

To confirm that the effects of Sema7A on GnRH nerve processes were mediated by PlexinC1 and its downstream effectors, we silenced *PlexinC1* in GnV3 cells using a construct coding for a *PlexinC1* short hairpin RNA (shPlxnC1, Fig. 4a–c) and analysed the effect of Sema7A on neurite growth (Fig. 4a,b). Under basal conditions, the addition of Sema7A to the culture medium induced a significant retraction (~70%) of neurites (Fig. 4b,c), which was not detected following PlexinC1 knockdown (Fig. 4a,b). Western blot experiments revealed that Sema7A-induced phosphorylation of FAK, ERK1/2 and cofilin is impaired in shPlxnC1-transfected cells as compared with mock-transfected cells (Fig. 4c).

The intracellular region of the plexins contains a guanosine triphosphatase (GTPase)–activating protein (GAP) domain for Ras and its homologues^40^. Recently, Wang *et al*.^41^ showed that semaphorin-induced dimerization stimulates plexin GAP activity, thereby locally inactivating Rap1 and enabling neurite retraction in rat cortical neurons. To determine whether Rap1 is involved in the PlexinC1-dependent retraction of GnV3 neurites, we cultured GnV3 cells that did or did not exogenously express the constitutively active mutant construct Rap1-V12, which is refractory to the effects of RapGAP^42^. Notably, Rap1-V12 overexpression prevented Sema7A-induced neurite retraction (Fig. 4a,b). These data prompted us to directly test whether Sema7A was responsible for Rap1 inactivation in GnV3 cells. To this aim, we measured Rap1 activity by an affinity assay in which Rap1-GTP was pulled down from lysates of GnV3 cells incubated or not with Sema7A (250 ng ml^−1^) for 1 min. When compared with untreated cells, Sema7A-stimulated cells displayed about 50% reduction of active Rap1-GTP (Supplementary Fig. 4b).

### Sema7A-induced GnRH neurite retraction is physiologically relevant

To evaluate the importance of Sema7A-PlexinC1 signalling in the control of adult reproductive function, adult female rats with regular 4-day oestrous cycles were treated with Sema7A, vehicle (saline) or Sema7A plus a recombinant decoy ectodomain of PlexinC1, which acts as a ligand-trap sequestering Sema7A and preventing its binding to endogenous receptors. The solutions were infused into the ME via a cannula connected to a subcutaneously implanted osmotic minipump for 7 days (Fig. 4d–f), as described previously^30^. Within few days of infusion, Sema7A-treated animals showed a disruption of regular oestrous cyclicity, with 100% of days in the diestrus phase and a concomitant reduction of days in proestrus as compared with vehicle-treated animals (Fig. 4d,e,g). When Sema7A was neutralized by pre-incubation with soluble PlexinC1, in equimolar ratio, before infusion, rats maintained regular oestrous cyclicity (Fig. 4f,g). Together with our *ex vivo* results, these data suggest that Sema7A in the adult female brain is required for the neuroendocrine control of the ovarian cycle.

**Figure 5:**
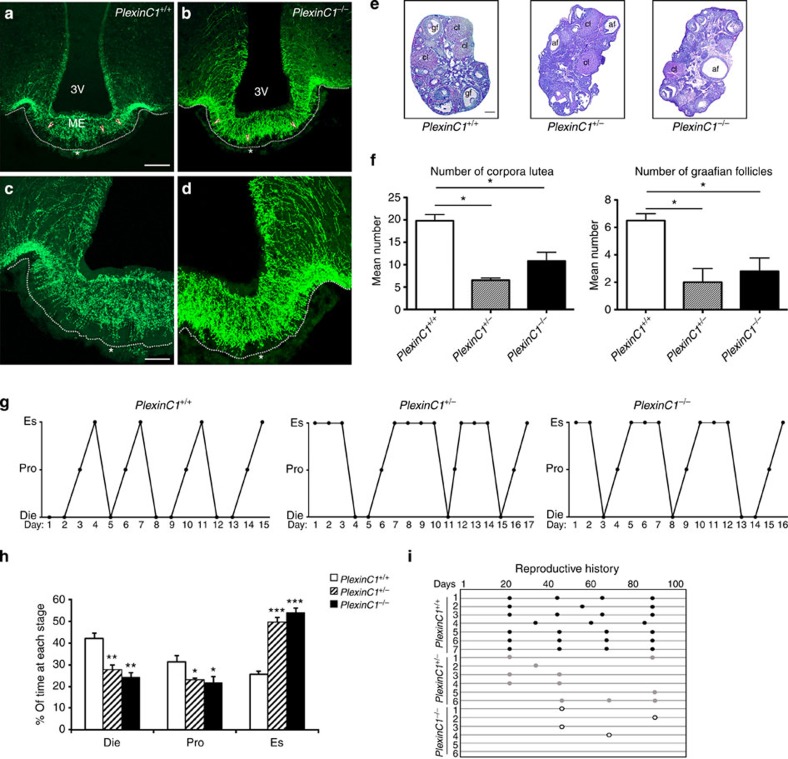
PlexinC1-deficient mice show abnormal GnRH innervation, ovulation, oestrous cyclicity and fertility. (**a**–**d**) Confocal photomicrographs showing GnRH immunoreactivity in coronal sections of hypothalamic ME in wild-type (**a**,**c**; *n*=7, females, 3–6 months old) and *PlexinC1*-null female mice (**b**,**d**; *n*=7, females, 3–6 months old). Arrowheads point to GnRH terminals and dotted lines define the basal lamina. Asterisks indicate the pituitary portal vessel bed in the external zone of the ME. (**e**) Morphological analysis of ovaries from 3- to 6-month-old control (*n*=5), *PlexinC1*^*+/−*^ (*n*=3) and *PlexinC1*^*−/−*^ (*n*=3) mice. Ovary sections (5 μm thick) were stained with haematoxylin–eosin. In both heterozygous and homozygous *PlexinC1* mice, the ovaries displayed a greater number of atretic follicles (af) and a relative paucity of corpora lutea (cl) and Graafian follicles (gf) as compared with wild-type littermates. (**f**) The numbers of corpora lutea and Graafian follicles were quantified in the ovaries of wild-type and mutant mice. Data are represented as means±s.e.m. Kruskal–Wallis test, *P<0.05. Scale bar, 100 μm (**e**). (**g**) Oestrous cyclicity in wild-type and *PlexinC1* mutant mice. Vaginal cytology was assessed for 15–17 days. (**h**) *PlexinC1*^*+/−*^ and *PlexinC1*^*−/−*^ animals spent significantly more days in the oestrous phase (Es) and less in diestrus (Die) and proestrus (Pro) as compared with control animals (*PlexinC1*^*+/+*^) (*n*=7 *PlexinC1*^*+/+*^, *n*=9 *PlexinC1*^*+/−*^, *n*=8 *PlexinC1*^*−/−*^, Kruskal–Wallis test, *P<0.05, **P<0.005, ***P<0.0005). (**i**) Schematic representation of the reproductive history of *PlexinC1*^*+/+*^ (*n*=7), *PlexinC1*^*+/−*^ (*n*=6) and *PlexinC1*^*−/−*^ (*n*=6) females. Black, grey and circled dots indicate litters produced by *PlexinC1*^*+/+*^, *PlexinC1*^*+/−*^ and *PlexinC1*^*−/−*^ mice, respectively, whereas X indicates an infertile female. Scale bars, (**a**,**b**) 100 μm, (**c**,**d**) 40 μm.

As Sema7A is responsible for GnRH terminal retraction in a PlexinC1-dependent manner, we next analysed whether the permanent genetic invalidation of PlexinC1 would result in the alteration of the GnRH innervation of the ME and/or in impaired fertility. Indeed, GnRH innervation in the ME of *PlexinC1*^*−/−*^ female mice was significantly more robust than in wild-type littermates (Fig. 5a–d). We then examined the fertility of 3- to 6-month-old *PlexinC1*^*+/+*^, *PlexinC1*^*+/−*^ and *PlexinC1*^*−/−*^ mice. In contrast to the ovaries of control mice, which contained large Graafian follicles and several corpora lutea, a histological inspection of the ovaries of adult *PlexinC1*^*+/−*^ and *PlexinC1*^*−/−*^ littermates revealed a significant reduction in the number of corpora lutea and Graafian follicles, indicating a reduced number of ovulations in mutant mice (Fig. 5e,f). The daily inspection of vaginal cytology in these mice over a period of 2 weeks revealed an absence of normal oestrous cyclicity, which was largely due to increased duration of estrus in *PlexinC1*^*+/−*^ and *PlexinC1*^*−/−*^ animals as compared with wild-type females (Fig. 5g,h).

The observation that *PlexinC1* mutant mice displayed altered GnRH innervation in the ME and abnormal ovulation and oestrous cyclicity suggested that the fertility of these mice could be reduced. We therefore examined fertility in *PlexinC1*^*+/+*^, *PlexinC1*^*+/−*^ and *PlexinC1*^*−/−*^ female mice using a continuous mating protocol for 90 days. Control mice had an average of 3.6±0.2 litters during this period. The number of litters in heterozygous female mice was 1.8±0.3, while homozygous knockout female mice had an average of only 0.6±0.2 litters during this period, confirming the reduction in the number of ovulations (Fig. 5i; *n*=6; one-way analysis of variance (ANOVA), *P*<0.0001; Tukey’s all pairs comparison: *PlexinC1*^*+/+*^ versus *PlexinC1*^*+/−*^, *P*<0.0001; *PlexinC1*^*+/+*^ versus *PlexinC1*^*−/−*^, *P*<0.0001; *PlexinC1*^*+/−*^ versus *PlexinC1*^*−/−*^ *P*<0.01).

### Sema7A-Itgb1 signalling in tanycytic end-foot expansion

As tanycytic end feet in the ME expand in response to Sema7A (Fig. 3a,b,d), we investigated *in vitro* which receptors’ signal transduction pathway is activated on Sema7A stimulation and explored the possibility that Sema7A could act in an autocrine/paracrine manner on these cells.

**Figure 6:**
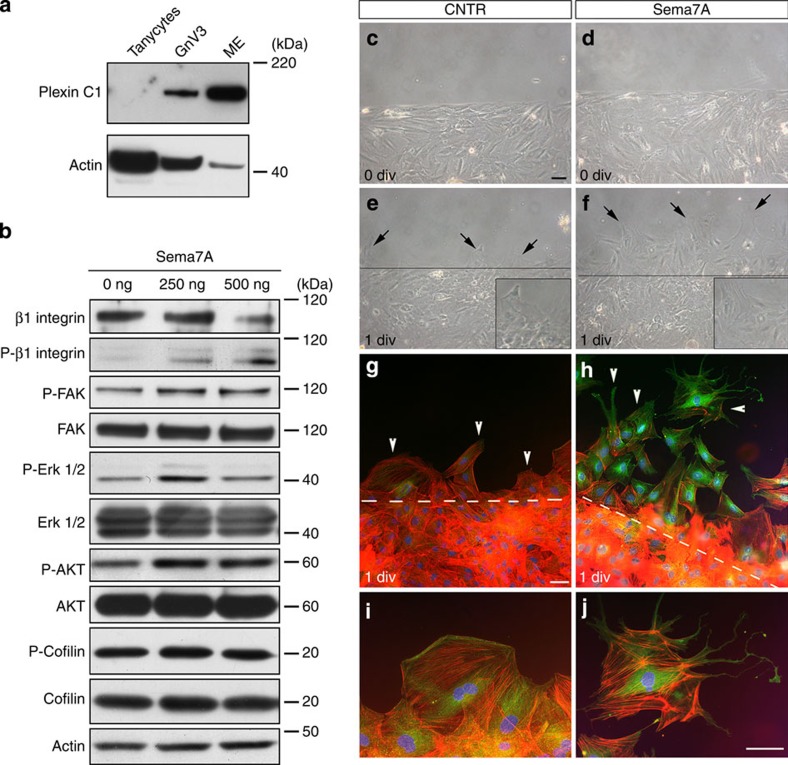
Sema7A promotes tanycytic end-feet growth through the Itgb1 signalling pathway. (**a**) Representative western blotting showing the expression of PlexinC1 and actin in primary tanycytes, in GnV3 cells and in median eminence (ME) lysates (positive control). Double amount of tanycytes cell lysates as compared with GnV3 protein extracts were intentionally loaded (see actin bands) to ascertain the lack of PlexinC1 expression in these cells. (**b**) Sema7A activates the Itgb1/MAPK pathway in primary tanycytes. Cells were treated with the indicated doses of recombinant Sema7A for 30 min and total cell lysates subjected to 3–8% SDS–PAGE and immunoblotting with the indicated antibodies. Experiments were done in triplicate with comparable results. (**c**–**j**) Representative photomicrographs of a scratch-wound-healing assay performed on primary tanycytes treated or not for 24 h with recombinant Sema7A (250 ng ml^−1^; *n*=5 independent experiments). Continuous (**e**,**f**) and dotted (**g**,**h**) lines indicate the borders of the scratch at the initial recording time. After 24 h of exposure to Sema7A, tanycytes invade a greater area of the scratch than in control cultures (**e**–**h**). (**g**–**j**) Immunolabelling of tanycytes for paxillin (green) and F-actin (red) at 1 day *in vitro* (div) after treatment shows that Sema7A induces cytoskeletal remodelling in tanycytes, which quickly develop end-feet-like structures (arrowheads, **h**) barely seen in control cells (arrowheads, **g**). Scale bars, (**c**–**f**) 30 μm, (**g**–**j**) 20 μm.

Our *in vivo* data demonstrate that vimentin-immunoreactive tanycytes do not express PlexinC1 (Supplementary Fig. 3i–l). To confirm the validity of our *in vitro* model, we first checked by western blotting the expression of PlexinC1 in primary tanycytes and in two positive controls, GnV3 cell line and ME tissues (Fig. 6a). PlexinC1 was strongly expressed in both positive controls but barely detectable or absent in tanycytes even when twice as much protein extract was loaded as compared with GnV3 cells (Fig. 6a).

Western blot analyses were then carried out to determine the expression of the other Sema7A receptor Itgb1 and the activation of its signal transduction complexes following Sema7A treatment (250 and 500 ng ml^−1^). These experiments showed that tanycytes expressed high levels of Itgb1, and that Sema7A induced the phosphorylation of Itgb1 and its intracellular downstream effector molecules FAK, ERK1/2 and AKT (Fig. 6b), but did not affect the phosphorylation of cofilin, a known downstream molecules of PlexinC1 (ref. 14).

To determine whether Sema7A could induce, also *in vitro*, cytoskeletal remodelling and tanycyte processes expansion, we inflicted a scratch wound on primary tanycytes and treated the cells for 24 h with Sema7A (250 ng ml^−1^). Exposure to Sema7A improved the rearrangement of the cells, resulting in shorter healing times than in untreated controls (Fig. 6c–f). The cultures were fixed and labelled for F-actin (using phalloidin) and paxillin (Fig. 6g–j). Sema7A stimulation induced extension of tanycytic processes, which also displayed strong immunoreactivity for paxillin (Fig. 6g,h; arrowheads), consistent with its involvement in the dynamic adhesion required for cell protrusion.

These results show that Sema7A induces cytoskeletal remodelling in tanycytes, in keeping with end-foot expansion observed *in vivo*, probably through the activation of the Itgb1/FAK/mitogen-activated protein kinase (MAPK) pathway.

### Itgb1 function in normal GnRH secretion and ovulation

**Figure 7:**
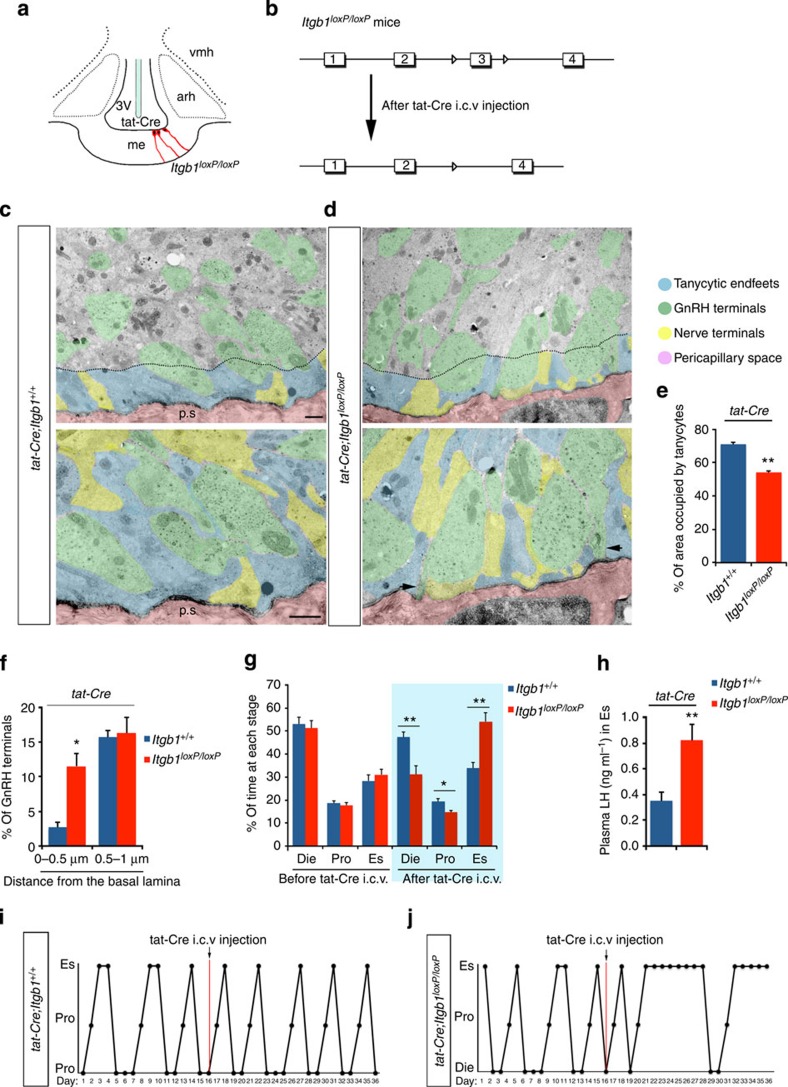
Itgb1 silencing in tanycytes of the ME disrupts neuroglial functional plasticity, oestrous cyclicity and LH levels. (**a**) Targeted Tat-cre delivery into the third ventricle of *Itgb1*^*loxP/loxP*^ and wild-type littermates *Itgb1*^*+/+*^. (**b**) Exon 3 of the *Itgb1* gene is flanked by two LoxP sites and is excised after cre recombination. Animals (3–6 months old) were killed 20 days after injection and ME was dissected and processed for electron microscopy. (**c**,**d**) Representative electron micrographs of GnRH-immunoreactive axon terminals (immunogold, green), hypothalamic nerve terminals (yellow) and tanycytic end feet (highlighted in blue) from proestrus *Tat-cre;Itgb1*^*+/+*^ and *Tat-cre;Itgb1*^*loxP/loxP*^ mouse hypothalamic explants containing the ME. (**d**) Itgb1 removal in tanycytes induces tanycytic end-feet to retract from the pericapillary space (p.s., pink) and facilitates the sprouting of GnRH nerve terminals towards the pericapillary space (arrows). (**e**) Quantitative analysis of the percentage of area occupied by tanycytes in the external zone of the ME at <2 μm from the pericapillary space in explants from mice in proestrus in control and *Itgb1*-deleted mice (*n*=6 *Tat-cre;Itgb1*^*+/+*^, *n*=8 *Tat-cre;Itgb1*^*loxP/loxP*^, Wilcoxon–Mann–Whitney test, ^**^P<0.005). (**f**) Quantitative analysis of the percentage of GnRH nerve terminals located between 0–0.5 μm and 0.5–1 μm from the pericapillary space in the external zone of the ME, in proestrus explants from control and *Itgb1*-deleted mice (*n*=6 *Tat-cre;Itgb1*^*+/+*^, *n*=4 *Tat-cre;Itgb1*^*loxP/loxP*^, Wilcoxon–Mann–Whitney test, *P<0.05). (**g**) Oestrous cyclicity of *Tat-cre;Itgb1*^*loxP/loxP*^ and *Tat-cre;Itgb1*^*+/+*^ animals was assessed for 16 days before Tat-cre injection and 20 days after injection in *Tat-cre;Itgb1*^*loxP/loxP*^ (*n*=12 *Tat-cre;Itgb1*^*+/+*^, *n*=10 *Tat-cre;Itgb1*^*loxP/loxP*^, Wilcoxon–Mann–Whitney test, *P<0.01, ^**^P<0.005). (**h**) Plasma LH levels in *Tat-cre;Itgb1*^*loxP/loxP*^ and in *Tat-cre;Itgb1*^*+/+*^ mice (*n*=8 *Tat-cre;Itgb1*^*+/+*^, *n*=7 *Tat-cre;Itgb1*^*loxP/loxP*^, unpaired Student’s *t*-test, ^**^P<0.005). Data are represented as means±s.e.m. (**i**,**j**) Representative oestrous cycle profiles of *Tat-cre;Itgb1*^*+/+*^ and *Tat-cre;Itgb1*^*loxP/loxP*^ animals. Scale bars, (**d**) 1 μm.

Given that Sema7A signalling engaged Itgb1 activation in primary tanycyte cultures (Fig. 6b), we next examined the involvement of the Itgb1 signalling pathway in the cyclic morphological changes of tanycytic end feet in the ME of adult mice, potentially contributing to regulate the coupling between GnRH nerve terminals and blood vessels. We used the injection of Tat-cre into the brain of *Itgb1*^*loxP/loxP*^ mice (Fig. 7a,b) to generate *Tat-cre;Itgb1*^*loxP/loxP*^ mice, in which *Itgb1* (the β1-integrin gene) was selectively knocked out in tanycytes. Hypothalamic MEs were acutely isolated from *Itgb1*^*+/+*^ and *Itgb1*^*loxP/loxP*^ female mice 20 days after Tat-cre stereotaxic injection, fixed and processed for electron microscopy using 15 nm gold-particle labelling to visualize GnRH terminals. Electron microscopic analyses of explants containing the ME from preovulatory control mice in proestrus (Fig. 7c,d) revealed the presence of several hypothalamic (yellow) and GnRH (green) nerve terminals in close proximity to the pericapillary space (pink; Fig. 7c). Following the conditional removal of Itgb1 in tanycytes (Fig. 7d), the area occupied by tanycytic end feet (blue) at <2 μm from the pericapillary space was significantly reduced by 20% as compared with controls (Fig. 7e), with a corresponding increase in the space occupied by nerve terminals (Fig. 7c,d).

Intriguingly, even though many GnRH nerve terminals were found to be in close proximity to the parenchymatous basal lamina in proestrus control explants (*Tat-cre;Itgb1*^*+/+*^), no direct contact between GnRH axons and the vascular wall was seen in this group (Fig. 7c). However, in the *Tat-cre;Itgb1*^*loxP/loxP*^ group, we frequently observed GnRH terminals sprouting and making direct contacts with the basal lamina of the ME (Fig. 7d, arrows) in areas in which tanycyte processes engulfing GnRH nerve terminals were much less conspicuous. Quantitative analysis of the distance of GnRH nerve processes from the pericapillary space confirmed that the percentage of GnRH-immunoreactive terminals located in tight proximity with the basal lamina (between 0 and 0.5 μm from the pericapillary space) was significantly increased in *Tat-cre;Itgb1*^*loxP/loxP*^ mice as compared with control animals, whereas it did not change at a distance between 0.5 and 1 μm (Fig. 7f).

These data show that the cell-specific removal of Itgb1 promotes tanycytic end-foot retraction in this region, making the pericapillary space more accessible to GnRH nerve terminals.

We then studied the physiological significance of Sema7A- Itgb1 signalling in tanycytes by analysing the oestrous cycle in these conditional knockout mice. We detected significant alterations in oestrous cyclicity shortly after Tat-cre injection (Fig. 7g). Similar to *PlexinC1* knockout mice, *Tat-cre;Itgb1*^*loxP/loxP*^ animals spent more days in the oestrous phase and correspondingly less in diestrus and proestrus than control animals (*Tat-cre;Itgb1*^*+/+*^) (Fig. 7g,i,j).

To examine the endocrine milieu contributing to abnormal cycles in *Tat-cre;Itgb1*^*loxP/loxP*^ mice, we measured LH as an index of GnRH release *in vivo*. In adult *Tat-cre;Itgb1*^*loxP/loxP*^ female mice on estrus, serum LH levels were abnormally elevated compared with the levels seen in control *Tat-cre;Itgb1*^*+/+*^ females (Fig. 7h). Pathologically elevated LH plasma concentrations have been previously reported in a reproductive neuroendocrine model of polycystic ovary syndrome-like mice^43^.

These data suggest that removal of Itgb1 from tanycytes reprogrammed the output of the hypothalamic–pituitary–gonadal axis in adults, confirming the increased secretion of GnRH into the pituitary portal blood vessels in the absence of tanycytic end-foot expansion.

## Discussion

Over the past two decades, it has become clear that GnRH terminals of the ME undergo dynamic transformations as a function of gonadectomy^20^ as well as of fluctuating physiological conditions that influence the distance between GnRH terminals and the basal lamina^19^. However, the molecular cues responsible for these dynamic morphological changes have not been elucidated so far.

Recent evidences suggest that the semaphorins continue to be expressed in the postnatal brain and may have important implications for neuronal plasticity and nervous system physiology^14^. In recent years, there has been increasing interest in the potential influence of semaphorins on the development and homeostasis of hormone systems and, conversely, on how circulating reproductive hormones could regulate semaphorins’ expression^44^.

Here we show that in the ME the tanycytic expression of Sema7A varies as a function of the hormonal state of the animal during the oestrous cycle, being maximal in the afternoon of diestrus II. We propose that Sema7A released by hypothalamic tanycytes cyclically induces GnRH neurons to retract their terminals away from the pericapillary space through PlexinC1 signalling and concomitantly promotes tanycytic end-feet expansion via Itgb1 activation, making the pericapillary space inaccessible to GnRH nerve terminals. This mechanism regulates neuropeptide release at key stages of the ovarian cycle, such as at diestrus, when GnRH secretion into the portal circulation is low.

These results complement a recent study in which we demonstrated the important role played by another semaphorin, Sema3A, on the GnRH system at a different stage of the oestrous cycle^45^. We indeed showed that the rise of oestrogen levels in proestrus triggers the release of the 65-kDa isoform of Sema3A (p65-Sema3A) by the endothelial cells of the ME, which in turn promotes GnRH axonal elongation towards the vascular plexus to facilitate GnRH release into the portal blood and thus modulate the amplitude of the preovulatory LH surge^45^. Whereas in the present study we suggest that in diestrus, when P4 secretion reaches peak values (35 ng ml^−1^)^31^,^46^ while oestrogen levels are low (5 pg ml^−1^)^31^,^46^, P4 stimulates Sema7A expression in tanycytes and the Sema7A-mediated expansion of tanycytic end feet, which ensheathe GnRH nerve terminals and thus prevent free diffusion of the neurohormone into the percapillary space (Figs 1b and 2d).

Altogether, these studies shed light on the molecular mechanisms responsible of the progression of the oestrous cycle in rodents and suggest that this phenomenon relies, at least in part, on the antagonistic effects of two ME semaphorins whose expression is periodically influenced by circulating sex steroids, estradiol (E2) and progesterone (P4), respectively.

It is tempting to speculate that, in mammalian species in which P4 has been shown to terminate the GnRH/LH surge^47^,^48^, P4 may arrest GnRH release by promoting the Sema7A-mediated engulfment of GnRH nerve terminals by tanycytic end feet.

Importantly, these experiments were never carried under non-physiological ‘progesterone-only’ conditions, as ovariectomy does not completely abolish circulating oestrogens. In fact, oestrogens are also produced in smaller amounts by other tissues such as the liver, adrenal glands and breasts (in rodents as well as in humans)^49^,^50^. Moreover, we and others have shown that in OVX rats, circulating oestrogens are still present at low levels (8.1±1.6 pg ml^−1^)^51^,^52^, reminiscent of the diestrus state (between 5 and 15 pg ml^−1^)^31^,^46^.

The physiological state of the female depends not on the presence or absence, or even the levels of circulating ovarian hormones *per se*, but on the ratio of progesterone to oestrogens^53^. Thus, given the continued presence of small amounts of extraovarian oestrogens even after ovariectomy, OVX+P4 animals experience a high P4:E2 ratio, reminiscent of diestrus, whereas in the OVX+E2 or OVX+E2+P4 situations where exogenous oestrogens are reintroduced, the P4:E2 ratio is much lower, mimicking conditions during proestrus, when the preovulatory LH surge occurs.

We also found a causal interaction between the expression patterns of TGF-β1 and Sema7A, with the former being upregulated in tanycytes in response to P4, and thereby upregulating Sema7A in the same cells. These findings are in agreement with previous studies showing that Sema7A expression is stimulated by TGF-β1 in non-neuronal tissues^26^ and, together with previous findings^54^, strongly suggest that tanycytes modulate the secretory activity of GnRH neurons via juxtacrine interactions involving TGF-β1 signalling and Sema7A-induced cytoskeletal rearrangements in GnRH neurons and tanycytes. Consistently, TGF-β1 mRNA expression in the ME FACS-isolated cells was differentially expressed during the oestrous cycle, being significantly higher in diestrus in both tanycytes (Tomato-positive) and Tomato-negative cells (astrocytes and endothelial cells). These data provide evidence that multiple cell types of the ME express TGF-β1 and modulate its expression in response to fluctuating levels of gonadal steroids, suggesting the existence of a feed-forward mechanism. Accordingly, previous studies showed that TGF-β1 is abundantly expressed in both glial cells and capillaries in the rodent’s ME^27^.

Sema7A has been previously shown to bind PlexinC1 (ref. 55) but no role was attributed to this interaction in neurons until now. However, the high-affinity interaction of Sema7A and PlexinC1 (ref. 55), together with their expression profile pattern^18^, indicate the possibility of functional cooperation between these proteins in neurons. In this work, we have shown that GnRH neurons have the ability to directly respond to fluctuating levels of tanycytic Sema7A through the activation of PlexinC1, resulting in the physiologically crucial remodelling of their processes.

We have previously shown that the expression pattern of the two Sema7A receptors in GnRH neurons appears to be spatiotemporally regulated: at early stages, migrating GnRH neurons only express Itgb1, whereas they begin to express PlexinC1 during subsequent developmental stages and in anatomical areas at which these cells stop migrating^15^. Moreover, we have demonstrated that the loss of Sema7A or the conditional inactivation of Itgb1 in GnRH neurons had impacts on the development of this system, resulting in the significant reduction of the GnRH neuronal population in the brain of adult mice, as well as reduced gonadal size and altered fertility^15^,^16^. An analysis of *PlexinC1*^*−/−*^ embryos has not revealed any difference in the migratory process of GnRH neurons, indicating that Sema7A signals through Itgb1 to regulate GnRH cell motility during development^15^. On the other hand, the current work provides compelling evidence that PlexinC1 is required to mediate the effects of Sema7A on mature (adult) GnRH neurons.

Using GnRH cell lines, we have also demonstrated that Sema7A rapidly induces actin cytoskeletal remodelling that leads to the retraction of GnRH processes in a PlexinC1-dependent manner. Whether similar phenomena actually occur in GnRH nerve terminals *in vivo* is not known; however, our *in vitro* experiments suggest that Sema7A activates the FAK–MAPK pathway and inactivates cofilin via PlexinC1 signal transduction pathway in GnRH neurons. Gene knockdown experiments indicate that Sema7A-PlexinC1 binding triggers a signalling cascade that leads to cofilin phosphorylation and cytoskeletal remodelling, leading to axonal retraction. In support of these findings, FAK–MAPK activation and the phosphorylation of cofilin have previously been linked to Sema3A-induced growth-cone collapse^56^,^57^. However, information regarding the signalling pathways stimulated by PlexinC1 is fairly limited, even though in other non-neuronal cell types, Sema7A binding to PlexinC1 can induce the activation of LIM kinase II and inactivation of cofilin, resulting in decreased cell adhesion and migration^38^,^39^.

The identification of the RasGAP domain in the plexins more than a decade ago raised the critical question of which Ras family member they acted on for signal transduction^58^,^59^. Recently, Wang *et al.*^41^ have demonstrated that the purified cytoplasmic region of the plexins uses a non-canonical catalytic mechanism to act as a GAP for Rap, but not for R-Ras or M-Ras. In the same work, they have also shown that among the plexins tested, the highest GAP activity for Rap1 is displayed by the purified cytoplasmic region of PlexinC1. Consistent with these findings, we have shown here that the Sema7A-induced neurite retraction of GnRH neurons depends on the inactivation of Rap1. As the F-actin-severing protein cofilin is a downstream target of the Rap GTPases and the activation of this Rap–cofilin module is essential for cytoskeletal changes crucial to B- and T-cell function^60^, we hypothesize that Sema7A could promote neurite retraction in GnRH neurons through a similar PlexinC1-mediated inactivation of Rap1 and cofilin.

Using primary cultures of tanycytes, this study also highlights how tanycytes remodel in response to Sema7A- Itgb1 signalling, and the results obtained, notwithstanding their limitation inherent to the use of an *in vitro* model system, further substantiates the idea that the signalling pathways and effects of individual guidance molecules vary as a function of cellular context.

The functional consequences of the selective removal of Itgb1 in tanycytes of adult mice confirms that Itgb1 is required for the expansion of tanycytic end feet and normal reproductive cyclicity. Interestingly, this deletion leads to an alteration of the oestrous cycle with a predominance of oestrous stages with elevated circulating levels of LH. These mutant mice appear to recapitulate, at least in part, the reproductive neuroendocrine phenotype of the prenatal androgen-induced mouse model of polycystic ovary syndrome^43^. Indeed, Sullivan and Moenter^43^ have previously shown that prenatal androgen-induced females exhibit pathologically elevated levels of LH and a lengthening of the oestrous cycles with an increased duration of estrus. Our data thus suggest that alteration of the morphological plasticity of tanycytes during the oestrous cycle could play important and unexpected roles in the aetiology of some forms of hypothalamic infertility. On the same line, recent findings showing that gonadal steroids promote structural changes in the hypothalamus of young women during the menstrual cycle^61^ and that mutation in the *SEMA7A* gene can be found in patients with congenital hypogonadotropic hypogonadism^62^ suggest that our results could be of clinical relevance and may pave the way for the development of new treatment strategies in the central loss of reproductive competence in human syndromes and/or of new contraceptive strategies.

**Figure 8:**
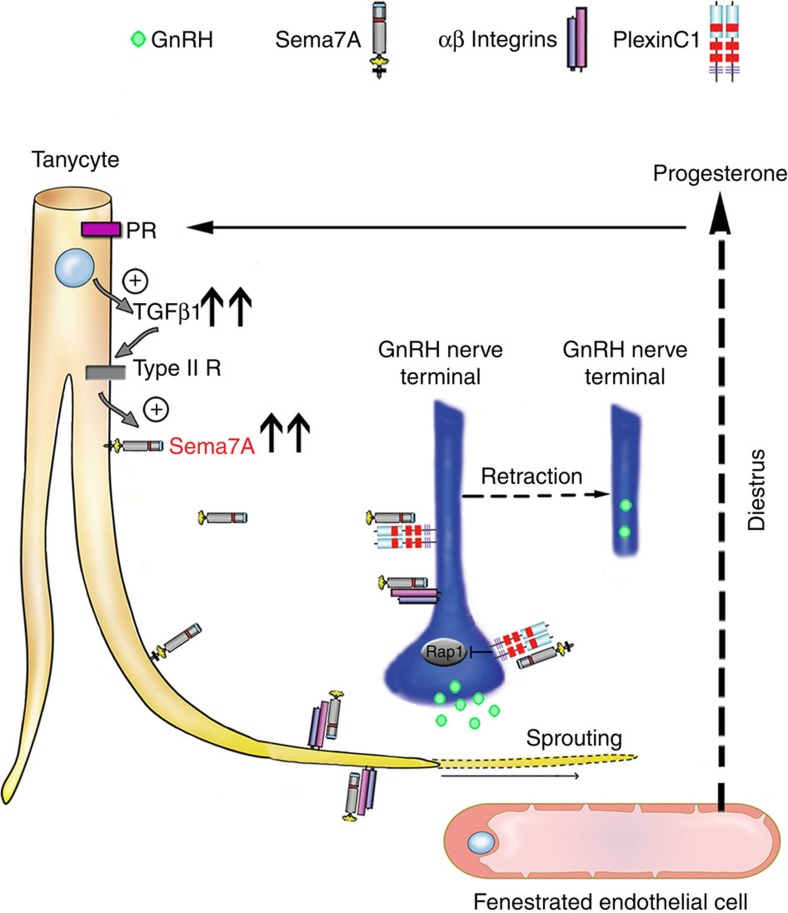
Schematic of the Sema7A-action in the ME of the hypothalamus. Schematic representation of the bifunctional mechanism of action of Sema7A on tanycytes (yellow cells) and GnRH terminals (blue) as a function of the oestrous phase. During diestrus, progesterone binds to its specific receptors, PR-A/B in tanycytes. Tanycytes then overexpress TGF-β1, which is responsible for the rise in Sema7A expression in tanycytes. Sema7A induces the elongation of tanycytic end feet through Itgb1 activation and retraction of GnRH nerve endings through the engagement of PlexinC1 and inactivation of Rap1 in GnRH terminals.

All together, these data uncover a hitherto unknown physiological role for tanycytes in the central control of fertility and concur to validate the bifunctional nature of semaphorins, here mediated by the differential engagement of PlexinC1 and Itgb1 as alternative Sema7A receptors in distinct cell types (Fig. 8). In addition, they enlighten the role of the semaphorin signalling pathways in ependymoglial–neuronal plasticity, which preserves homeostatic set points important for the survival of the individual and the species.

## Methods

### Animals

All animals were housed under specific pathogen-free conditions in a temperature-controlled room (21 °C –22 °C) with a 12 h light/dark cycle. Vaginal smears were examined daily and only rats that exhibited at least two consecutive 4-day oestrous cycles were used for experiments. Diestrus I and II were defined by the predominance of leukocytes in the vaginal lavage, the day of proestrus by the predominance of nucleated round epithelial cells and the day of estrus by large numbers of clustered cornified squamous epithelial cells.

Sprague–Dawley female rats (3–4 months old; Charles River, USA) were provided *ad libitum* access to water and standard laboratory chow (Special Diet Services, RM3, France, for mice; Purina lab chow, 5001, USA, for rats). *tdTomato*^*loxP/+*^ and *Itgb1*^*LoxP/LoxP*^ mice (breeding pairs 3–6 months old), in which exon three of Itgb1 is flanked by loxP sites, were purchased from the Jackson Laboratories (Bar Harbor, ME). *PlexinC1* and *Sema7A* mutant mice have been previously characterized^63^. Animal use was in compliance with Inserm guidelines for the care and use of laboratory animals and the European Communities Council Directive of 24 November1986 (86/609/EEC) regarding mammalian research, and approved by the Institutional Animal Care and Use Committee of Lille.

### Drugs

Recombinant Sema7A (1835-S3) and soluble PlexinC1 (5375-PC) were purchased from R&D Systems, MN, USA. TGFβ1 was purchased from Millipore (GF111). Progesterone for *in vitro* treatment was purchased from Sigma-Aldrich, USA (P6149). 17β-estradiol 3-benzoate (E8515), 17β-estradiol (E8875), progesterone (P0130) and sesame oil (S3547) were also purchased from Sigma-Aldrich.

### Ovariectomy and hormone treatments

Twenty cycling female rats (3- to 4-month-old females rats) were bilaterally ovariectomized (OVX, day 0) under anaesthesia by intraperitoneal injection of xylazine 10 mg kg^−1^ (Rompun 2%, Bayer) and ketamine 60 mg kg^−1^ (Ketalar, Parke-Davis). Animals were divided in 4 groups: 16 animals were killed at 1400, h 17 days after ovariectomy without receiving any treatment; 4 animals received a single s.c. injection of estradiol benzoate (E2, 3 μg per rat) at 1000, h, 15 days after ovariectomy, and were killed at 1400, h 2 days later. Four animals received a single s.c. injection of progesterone (P4, 30 μg per rat) at 1000, h and were killed at 1400, h on day 17. Four rats were injected with E2+P4 and three with saline only, and killed at 1400, h on day 17. OVX, E2- and P-treated rats ME were used for western blot analysis to check the expression of Sema7A and its receptors.

### Infusion of Sema7A

Adult female rats with regular 4-day oestrous cycle were treated with the Sema7A (*n*=6), vehicle (*n*=6, PBS) or Sema7A+soluble PlexinC1 (*n*=6, 5375-PC; R&D Systems). They were chronically infused into the ME (bregma: −3.4 mm, 10.25 mm depth from the skull) through a stereotaxically implanted infusion cannula (Plastics One, Roanoke, VA) connected to a subcutaneously implanted mini-osmotic pump (model 1007D, flow rate 0.5 μl h^−1^, capacity 100 μl, delivery period 7 days; Alzet, Palo Alto, CA). Each pump was loaded with sterile PBS (Invitrogen) containing Sema7A (0.25 μg μl^−1^ final) or Sema7A and PlexinC1 (0.25 μg μl^−1^). After connection to the infusion device and overnight priming in 0.9% NaCl at 37 °C, the assembly was implanted into cycling 190–200 *g* female rats. Oestrous cycle was monitored before and during surgery. Subsequent to the infusion experiment, animals were killed, to assess the implantation site of the cannula and check exhaustion of the infusion solution. MEs were collected, snap frozen in dry ice and store at −80 °C. To determine whether infused molecules actually targeted the ME, animals were killed 7 days after the initiation of the treatment, the brains were dissected and the sections of 20 μm thickness from the ME region of hypothalamus were cut and subjected to immunohistochemistry for GnRH staining.

### Tat-cre injection

*Itgb1*^*loxP/loxP*^ female mice (3–6 months old, numbers of animals used and corresponding genotypes are specified in the figure legends) and *tdTomato*^*loxP/+*^ female mice (3–6 months old, *n*=5) were placed in a stereotactic frame (Kopf Instruments, California) under anaesthesia (isoflurane) and a burr hole was drilled 1.7 mm posterior to the Bregma. A 10-μl Hamilton syringe was slowly inserted into the third ventricle (5.6-mm deep relative to the dura) and 1.5 μl of Tat-cre (2.1 mg ml^−1^) was injected using an infusion pump over 5 min. The Tat-cre fusion protein produced as detailed previously^23^.

### Tanycyte cell culture

Tanycyte cell cultures were performed as previously described^29^,^30^. Primary cultures of tanycytes were prepared using MEs dissected from 10-day-old rats. After a growing period of 8–10 days in 75 cm culture flasks containing DMEM-F-12 high-glucose medium supplemented with 10% calf serum and 2 mM L-glutamine, the astrocytes and tanycytes were isolated from contaminant cells by overnight shaking at 250 r.p.m. and were plated either in 15 cm dishes (1–1.5 million cells per dish) for immunoblot analysis. For immunohistochemistry, the cells were seeded in six-well plates on poly-L-ornithine (100 μg ml^−1^)- and laminin (2 μg ml^−1^)-coated coverslips. After reaching 90% confluence, the medium was replaced with a serum-free, astrocyte- and tanycyte-defined medium consisting of DMEM devoid of phenol red, supplemented with 2 mM L-glutamine, 15 mM HEPES, 5 μg ml^−1^ insulin and 100 μM putrescine. The cells were used 48 h later for experiments.

### Immunohistochemistry

Animals were anaesthetized with 200 mg kg^−1^ ketamine, perfused transcardially with 5–10 ml of saline, followed by 500 ml of 4% paraformaldehyde in 0.1 M phosphate buffer (PB), pH 7.4. Brains were removed and immersed in the same fixative for 2 h at 4 °C and stored in PB until slicing. Thirty-five micrometres of free-floating coronal sections containing the ME region were cut on a vibratome (VT1000S; Leica, Wetzlar, Germany) and collected in ice-cold PB and blocked in PBS with 5% normal donkey serum (D9663; Sigma) and 0.3% Triton X-100 (Sigma) for 1 h at RT before incubation with different primary antibodies (mentioned in antibodies index below), and then washed extensively in PBS and exposed to corresponding secondary antibodies (for details, see antibodies index for immunohistofluorescence) tagged with Fluor 488-conjugated (1:500), AlexaFluor 350 (1:500) and AlexaFluor 568 (1:500), for 1 h in 5% normal donkey serum. After washes, slices were incubated for 2 min with 0.02% Hoechst 33258 (H3569; Invitrogen) in PBS for fluorescent nuclear staining, and mounted on glass slides and coverslipped with Permafluor medium (434990; Immunon, Pittsburgh, PA). Control sections were incubated in the absence of a primary antibody and on brain sections of deficient mice (*Sema7A* and *PlexinC1* knockout animals, 3- to 6-month-old female mice, *n*=3). For these latter experiments, coronal sections containing the ME were incubated for 24 h at 4 °C in Sema7A (goat polyclonal; 1:400; AF1835; R&D Systems) or PlexinC1 (sheep polyclonal; 1:400; AF5375; R&D Systems) primary antibodies diluted in 0.01 M PBS, pH 7.4, 0.5% TritonX-100, and 1% normal serum, made in the same host species of the secondary antibody. For the avidin-biotin-peroxidase method, sections were then incubated for 1 h at room temperature (RT) in the appropriate secondary antibody (biotynilated horse anti-goat IgG, or biotynilated rabbit anti-sheep IgG; Vector Laboratories, Burlingame, CA) diluted 1:250 in 0.01 M PBS, pH 7.4, followed by the avidin-biotin-peroxidase complex (1:150; Vector Laboratories). To reveal immunoreactivity, we used 0.015% 3,3′-diaminobenzidine (DAB) and 0.0024% H_2_O_2_ in 0.05 M Tris-HCl, pH 7.6. After adhesion on gelatin-coated glass slides, sections were dehydrated and mounted in Sintex (Nuova Chimica, Cinisello Balsamo, Italy).

Sections were examined using an Axio Imager.Z1 ApoTome microscope (Carl Zeiss, Germany) equipped with a motorized stage and an AxioCam MRm camera (Zeiss). For confocal observation and analyses, an inverted laser scanning Axio observer microscope (LSM 710, Zeiss) with an EC Plan NeoFluor × 100/1.4 numerical aperture (NA) oil-immersion objective (Zeiss) was used (Imaging Core Facility of IFR114 of the University of Lille 2, France).

### Antibodies used for immunohistofluorescence experiments

The rabbit polyclonal anti-GnRH (1:3,000) was a generous gift from Professor G. Tramu (Centre Nationale de la Recherche Scientifique, URA 339, Université Bordeaux I, Talence, France).

Antibodies were against Sema7A (goat polyclonal; 1:400; AF1835; R&D Systems), PlexinC1 (sheep polyclonal; 1:400; AF5375; R&D Systems), β1-Integrin (rabbit polyclonal; 1:500, sc-8978; Santa Cruz), vimentin (chicken polyclonal, 1:2,000, AB5733, Millipore), progesterone receptors A/B (rabbit polyclonal, 3176S; 1:1,000, Sigma) and BSL-1 (endothelial cell marker, TRITC-labelled Bandeiraea Simplicifolia Lectin BS-1 L5264, Sigma). A donkey anti-chick AlexaFluor 568-conjugated secondary antibody (1:500) was used for vimentin, the donkey anti-rabbit AlexaFluor 488-conjugated secondary antibody (1:500) for Sema7A and the donkey anti-mouse AlexaFluor 350-conjugated secondary antibody (1:500) for GnRH detection. Phalloidin (1:100) used to stain actin cytoskeleton of fixed and permeabilized cells was labelled with rhodamine and purchased from Invitrogen (Eugene, OR).

### Isolation of hypothalamic tanycytes using fluorescence activated cell sorting

MEs from Tat-cre-injected *tdTomato*^loxP/+^ female mice (3–6 months old, *n*=5) fed *ad libitum* were microdissected and enzymatically dissociated using a Papain Dissociation System (Worthington, Lakewood, NJ), to obtain single-cell suspensions. FACS analysis was performed using an EPICS ALTRA Cell Sorter Cytometer device (Beckman Coulter, Inc.). The sorting decision was based on measurements of tdTomato fluorescence (excitation: 488 nm; detection: bandpass 675±20 nm) by comparing cell suspensions from tdTomato-positive and wild-type animals. For each animal, 5,000–8,000 tdTomato-positive cells were sorted directly into 10 μl extraction buffer: 0.1% Triton X-100 (Sigma-Aldrich) and 0.4 U μl^−1^ RNase OUT TM (Life Technologies).

### Quantitative RT–PCR analyses

For gene expression analyses, mRNAs obtained from FACS-sorted tanycytes were reverse transcribed using SuperScript III Reverse Transcriptase (Life Technologies) and a linear preamplification step was performed using the TaqMan PreAmp Master Mix Kit protocol (P/N 4366128, Applied Biosystems). Real-time PCR was carried out on an Applied Biosystems 7900HT Fast Real-Time PCR System, using exon-boundary-specific TaqMan Gene Expression Assays (Applied Biosystems) as follows: *DARPP32* (Ppp1r1b: Mm00454892_m1), *ERα* (*Esr1:* Mm00433149_m1), *ERβ* (*Esr2:* Mm00599821_m1), *PR-A/B* (*Pgr:* Mm00435628_m1), *TGF-β1* (*Tgfb1:* Mm01178820_m1) and *Sema7a* (*Sema7a:* Mm01171202_m1). Control housekeeping genes were *r18S* (18S: Hs99999901_s1) and *ACTB* (Actb: Mm00607939_s1). Gene expression data were analysed using SDS 2.4.1 and Data Assist 3.0.1 software (Applied Biosystems).

### Immortalized GnRH cell line

GnV-3 cells are 1 of the 11 clones of GnRH-expressing cells obtained by the conditional immortalization of adult rat hypothalamic cell cultures^34^. GnV-3 cells express markers of well-differentiated neurons and do not express markers of glial cells^35^. Cells were grown in proliferation medium consisting of Neurobasal A medium with B27 supplement (20 μl ml^−1^, Invitrogen-Gibco), PSN (1X, Invitrogen), Glutamax I (Invitrogen), Doxycycline hydrochloride (0.5 μg ml^−1^, Sigma), FBS (10 μl ml^−1^, Biological Industries) and βFGF (5 ng ml^−1^, Invitrogen). Doxycycline promotes the proliferation of these conditional immortalized cells. To induce their differentiation, the culture medium was replaced by differentiation medium (containing Neurobasal A, B27 supplement, PSN, and glutamax I and βFGF).

Short hairpin RNA for PlexinC1 and Rap1-V12 plasmids, employed for GnV3 transfections, were produced by Dr Tamagnone (Institute for Cancer Research at Candiolo, University of Torino, Italy).

### GnV3 morphological analysis

Changes in GnV3 cell morphology were evaluated by staining the cells with Alexa 588-X phalloidin (Molecular Probes, Eugene, OR) to visualize cytoskeletal actin. GnV3 cells were incubated overnight with or without a recombinant human Semaphorin-7A/Fc chimera (100–250 ng ml^−1^; 1835-S3, R&D Systems). Quantification of GnRH fibre length was performed by an experimentator, blind to culture treatments on digitized photomicrographs using the NeuronJ plugin of ImageJ software (National Institute of Health, Bethesda, USA).

### Immunoblotting

Female rats (*n*=4 independent experiments per oestrous cycle phase; 3–4 months old) were decapitated at 1600, h on the day of diestrus II (Di16h) or on proestrus or estrus at the same time (Pro16h). After rapid removal of the brain, the meninges and optic chiasma were removed and the ME was dissected under a binocular magnifying glass with Wecker’s scissors (Moria, France). After dissection, each fragment was placed in a microcentrifuge tube, snap frozen in liquid nitrogen and stored at −80 °C.

Protein extracts of each ME sample were prepared in 100 μl lysis buffer (pH 7.4, 25 mM Tris, 50 mM β-glycerophosphate, 1.5 mM EGTA, 0.5 mM EDTA, 1 mM sodium pyrophosphate, 1 mM sodium orthovanadate, 10 μg ml^−1^ leupeptin and pepstatin, 10 μg ml^−1^ aprotinin, 100 μg ml^−1^ phenylmethyl sulfonyl fluoride and 1% Triton X-100) by trituration of the fragments through 22- and 26-G needles in succession. The tissue lysates were cleared by centrifugation at 12,000*g* for 15 min and protein content was determined using the Bradford method (BioRad, Hercules, CA). We added 4 × sample buffer (Invitrogen) and 10 × reducing agent (Invitrogen) to the samples and boiled for 5 min before electrophoresis at 150 V for 75 min in precast 3%–8% SDS–polyacrylamide Tris-acetate gels according to the protocol supplied with the NuPAGE system (Invitrogen, Carlsbald, CA). When necessary, the samples were stored at –80 °C until use.

For primary and immortalized cell extracts, samples were boiled for 5 min after thawing and electrophoresed for 75 min at 150 V for 75 min in precast 3%–8% SDS–polyacrylamide Tris-acetate gels according to the protocol supplied with the NuPAGE system (Invitrogen). After size-fractionation, the proteins were transferred onto Nitrocellulose membranes (0.2 μm pore-size membranes; LC2002; Invitrogen) in the blot module of the NuPAGE system (Invitrogen) for 75 min at RT. Blots were blocked for 1 h in TBS with 0.05% Tween 20 (TBST) and 5% non-fat milk at RT, incubated overnight at 4 °C with their respective primary antibodies and washed four times with TBST before being exposed to horseradish peroxidase-conjugated secondary antibodies diluted in 5% non-fat milk-TBST for 1 h at RT. The immunoreactions were detected with enhanced chemiluminescence (NEL101, PerkinElmer, Boston, MA).

The goat polyclonal anti-actin (sc-1616; 1:1,000), mouse monoclonal anti-P-cofilin (sc271923; 1:500), rabbit anti-cofilin (sc33779; 1:500), rabbit anti-integrin β1 (sc8978), mouse monoclonal anti-P-FAK (sc81493; 1:1,000) and mouse monoclonal anti-FAK (sc56901; 1:500) antibodies were purchased from Santa Cruz Biotechnology (Santa Cruz, CA). The rabbit monoclonal anti-phospho-Akt (Ser473; 9275S; 1:1,000), rabbit monoclonal Akt (4691S; 1:1,000), rabbit polyclonal anti-phospho-p44/42 MAPK (p-Erk1/2) (Thr202/Tyr204; 9101L; 1:1,000), rabbit polyclonal anti-p44/42 MAPK (Erk1/2) (9102L; 1:1,000) and rabbit polyclonal anti-progesterone receptor A/B (3176S; 1:1,000) antibodies were purchased from Cell Signaling Technology (Beverly, MA, USA). Mouse anti-PlexinC1 (AF5375; 1:500) antibodies were purchased from R&D System (Beverly, MA, USA). Rabbit anti-P-β1 integrin (AB5189; 1:500), rabbit anti-paxillin (AB2264; 1:500), rabbit anti-Sema7A (ab23578; 1:500) and TGF-β1 (3176S; 1:500) antibodies were purchased from Abcam Technology (Beverly, MA, USA). Secondary antibodies used for western blotting detection (anti-mouse (1:8,000), anti-rabbit (1:10,000) and anti-goat/sheep (1:10,000), all horseradish peroxidase-conjugated) were purchased from Sigma (Saint-Quentin Fallavier, France).

Full images of western blottings are shown in Supplementary Figs 5–11.

### Rap1 pull-down assay

Rap1-GTP pull-down was performed with the Active Rap1 pull-down detection kit (Thermo), according to the manufacturer’s protocol. Briefly, GnV3 cells were grown for 3 days under differentiation condition^34^,^35^. Cells were then stimulated with 250 ng ml^−1^ Sema7A (R&D Systems) for 1 min and proteins were solubilized in a buffer provided with the kit (with protease inhibitors). The same amount of total proteins was incubated with GST-tagged Rap-binding domain of RalGDS (pre-loaded onto glutathione beads) to pull down active GTP-bound Rap1. Rap1 levels in the different samples were then assessed by western blotting analysis, by using Rap1 antibody (provided with the detection kit).

### Assessment of ultrastructural changes in the ME

To determine ultrastructural changes in GnRH nerve terminal plasticity, *ex vivo* experiments were carried out according to previously described protocols^19^,^31^. Female rats weighting 250–300 g (3–4 months old, *n*=3–5 animals/experimental condition) were killed on diestrus II or proestrus by decapitation. After rapid removal of the brain, hypothalamic explants were microdissected without damaging the MEs. Explants were placed in 12-well plates and preincubated for 30 min at 37 °C in 1 ml of Krebs–Ringer bicarbonate buffer, pH 7.4, containing 4.5 mg ml^−1^ D-dextrose and 5 μM tetrodotoxin, with or without recombinant human Semaphorin-7A/Fc chimera (1 μg ml^−1^; 1835-S3, R&D Systems), under an atmosphere of air containing 5% CO_2_. Explants were then processed for electron microscopy as described previously^19^,^31^. Briefly, tissues were fixed by immersion in a solution of 2% paraformaldehyde, 0.2% picric acid and 0.1% glutaraldehyde in 0.1 M phosphate buffer, pH 7.4, for 2 h at 4 °C. Tissues were postfixed with 1% OsO_4_ in phosphate buffer for 1 h at RT. After dehydration, tissues were embedded in Araldite. Semithin sections (1–2 μm thick) were used to progressively approach and identify the portion of the ME targeted for ultrastructural studies, that is, the area where the pituitary stalk becomes distinct from the base of the hypothalamus but still remains attached to it by the hypophyseal portal vasculature^19^. This area, which does not extend beyond 20 μm, contains high numbers of GnRH fibres. To detect GnRH immunoreactivity, ultrathin sections (80–90 nm thick) collected on Parlodion 0.8%/isoamyl acetate-coated 100 mesh grids (EMS, Fort Washington, PA) were treated using an immunogold procedure described previously^19^. Briefly, after a preliminary treatment with H_2_O_2_ (10%, 8 min) and a blocking step in TBS (0.1 M Tris, pH 7.4, 0.15 M NaCl) containing 1% normal goat serum and 1% bovine albumin serum (10 min at RT), the grids were floated on a drop of the following reagents and washing solutions: (1) rabbit anti-GnRH (1:10,000) in bovine albumin serum for 60 h at 4 °C, (2) TBS to remove excess antibodies (three times for 10 min), (3) colloidal gold (18 nm)-labelled goat anti-rabbit immunoglobulins (Jackson ImmunoResearch) 1:20 in TBS for 90 min at RT, (4) TBS (three times for 10 min) and (5) distilled water (three times for 10 min). The sections were then counterstained with uranyl acetate and lead citrate before observation. The specificity of the GnRH antisera used has been discussed previously^19^,^31^. Ultrathin immunolabelled sections were examined with a Zeiss transmission electron microscope 902 (Leo, Rueil-Malmaison, France) and images were acquired using a Gatan Orius SC1000 CCD camera (Gatan France, Grandchamp, France). The morphometric analysis was performed by an experimentator blind to the hypothalamic explant treatment on digitalized images taken at an original magnification of × 12,000 from 10 to 15 ultrathin sections per animal, with a space of 25 sections between them, to avoid taking the same GnRH nerve terminal into consideration twice (the diameter of a GnRH nerve terminal rarely exceeds 2 μm). All GnRH-immunoreactive nerve terminals located at <10 μm from the parenchymatous basal lamina (that is, the pial surface of the brain) were taken into consideration, that is, more than 100 distinct axon terminals per animals (that is, almost all GnRH nerve terminals abutting onto the pituitary portal blood vessels in the aforementioned 20-μm-thick region of the ME). Immunolabelled terminals confined to a distance of 10 μm or less from the basal lamina were imaged and the distance from the nerve terminal and the pericapillary space recorded. The number of immunoreactive GnRH terminals confined to a distance of <1 μm from the basal lamina was then counted for each animal and multiplied by 100 to obtain the percentage of GnRH terminals contacting the basal lamina in each treatment condition. The area occupied by tanycytic end feet at <2 μm from the basal lamina was also evaluated for each animal and under each treatment condition.

Similar electron microscopic analyses were performed in 3- to 6-month-old proestrus *Tat-cre;Itgb1*^*+/+*^ (*n*=6) and *Tat-cre;Itgb1*^*loxP/loxP*^ (*n*=8) mice.

### GnRH secretion determination

ME explants were dissected from female rats (3–4 months old, *n*=18) and processed as previously described^32^. Briefly, ME explants were incubated in artificial cerebrospinal fluid with the following composition (in mM): NaCl 117, KCl 4.7, NaH_2_PO_4_ 1.2, NaHCO_3_ 25, CaCl_2_ 2.5, MgCl_2_ 1.2, glucose 10, bubbled with 95% O_2_–5% CO_2_ (pH 7.4, osmolarity 304 mOsm). Medium was collected before and after explants were treated with or without recombinant human Semaphorin-7A/Fc chimera (1 μg ml^−1^; 1835-S3, R&D Systems), followed by incubation at 37 °C for 4 h at 300 r.p.m. Explants were treated with KCl 0.05 M at the end of the incubation period to confirm their viability. Collected media were analysed for GnRH content following a GnRH ELISA protocol (Phoenix Pharmaceuticals Inc., California, catalogue number FEK-040-02).

### Plasma LH assay

Plasma LH was measured using a Rodent LH ELISA kit (Endocrine Technologies, Newark, CA) with a sensitivity of 0.03 ng ml^−1^ and 7% intra-assay and 10% inter-assay coefficients of variance.

### Image analysis

For confocal observations and analyses, an inverted laser scanning Axio observer microscope (LSM 710, Zeiss) with EC Plan NeoFluor 10 × /0.3 NA, 20 × /0.5 NA and 40 × /1.3 NA (Zeiss) objectives were used (Imaging Core Facility of IFR114 of the University of Lille 2, France). ImageJ (National Institute of Health) and Photoshop CS5 (Adobe Systems, San Jose, CA) were used to process, adjust and merge the photomontages.

### Statistics

All analyses were performed using Prism 5 (GraphPad Software) and assessed for normality (Shapiro-Wilk test) and variance, when appropriate. Sample sizes were chosen according to the standard practice in the field. Data were compared by a two-tailed unpaired Student’s *t*-test, one-way ANOVA for multiple comparisons or by two-way repeated-measures ANOVA. A Tukey’s *post-hoc* test was performed when appropriate. The significance level was set at *P*<0.05. For comparison between two or multiple groups not having a normal distribution, the non-parametric tests unpaired test (Wilcoxon–Mann–Whitney) and Kruskal–Wallis, respectively, were used. A *P*-value<0.05 was considered to indicate a significant difference. Data groups are indicated as mean±s.e.m. The number of biologically independent experiments, *P*-values and sex of the animals are indicated in the figure legends.

## Author contributions

J.P. performed most of the experiments. A.M. performed the cell sorting and RT–PCR experiments. I.C., F.L. and J.P. performed the i.c.v. surgeries and immunohistochemistry. A.L. and S.G. performed some of the *in vitro* experiments on GnV3 cell lines. D.M. performed electron microscopy and analysis of ultrastructural rearrangement. E.B. and S.A.M. generated tanycyte cultures and S.A.M. performed RT–PCR analysis on tanycytes. F.P. provided us with GnV3 cell lines. G.C. performed the pull-down experiments on GnV3 cells. R.S. and S.D.M. performed immunohistochemistry on *PlexinC1* and *Sema7A*-deficient mice, and analysed the fertility of *PlexinC1* mutant animals. M.M. produced the Tat-Cre protein. R.J.P. provided the *Sema7A* and *PlexinC1* mutant mice and participated in some experimental design of the project. L.T. produced all the short hairpin RNAss used in this work, coordinated some of the molecular biology experiments and participated in some experimental design of the project. P.G. and V.P. designed the project and wrote the manuscript.

## Additional information

**How to cite this article:** Parkash, J. *et al.* Semaphorin7A regulates neuroglial plasticity in the adult hypothalamic median eminence. *Nat. Commun.* 6:6385 doi: 10.1038/ncomms7385 (2015).

## Supplementary Material

Supplementary Information Supplementary Figures 1-11.

## References

[b1] Christian C. A. & Moenter S. M. The neurobiology of preovulatory and estradiol-induced gonadotropin-releasing hormone surges. Endocr. Rev. 31, 544–577 (2010). 20237240 10.1210/er.2009-0023 PMC3365847

[b2] Gonzalez-Martinez D., Hu Y. & Bouloux P. M. Ontogeny of GnRH and olfactory neuronal systems in man: novel insights from the investigation of inherited forms of Kallmann's syndrome. Front. Neuroendocrinol. 25, 108–130 (2004). 15571757 10.1016/j.yfrne.2004.06.001

[b3] Prevot V. *et al.* Gonadotrophin-releasing hormone nerve terminals, tanycytes and neurohaemal junction remodelling in the adult median eminence: functional consequences for reproduction and dynamic role of vascular endothelial cells. J. Neuroendocrinol. 22, 639–649 (2010). 20492366 10.1111/j.1365-2826.2010.02033.x PMC3168864

[b4] Herbison A. E. Multimodal influence of estrogen upon gonadotropin-releasing hormone neurons. Endocr. Rev. 19, 302–330 (1998). 9626556 10.1210/edrv.19.3.0332

[b5] Moenter S. M., Chu Z. & Christian C. A. Neurobiological mechanisms underlying oestradiol negative and positive feedback regulation of gonadotrophin-releasing hormone neurones. J. Neuroendocrinol. 21, 327–333 (2009). 19207821 10.1111/j.1365-2826.2009.01826.x PMC2738426

[b6] Ronnekleiv O. K. & Kelly M. J. Diversity of ovarian steroid signaling in the hypothalamus. Front. Neuroendocrinol. 26, 65–84 (2005). 16009409 10.1016/j.yfrne.2005.05.001

[b7] Piet R., Boehm U. & Herbison A. E. Estrous cycle plasticity in the hyperpolarization-activated current Ih is mediated by circulating 17beta-estradiol in preoptic area kisspeptin neurons. J. Neurosci. 33, 10828–10839 (2013). 23804103 10.1523/JNEUROSCI.1021-13.2013 PMC6618493

[b8] Prevot V. *et al.* Function-related structural plasticity of the GnRH system: a role for neuronal-glial-endothelial interactions. Front. Neuroendocrinol. 31, 241–258 (2010). 20546773 10.1016/j.yfrne.2010.05.003

[b9] Garcia-Segura L. M., Lorenz B. & DonCarlos L. L. The role of glia in the hypothalamus: implications for gonadal steroid feedback and reproductive neuroendocrine output. Reproduction 135, 419–429 (2008). 18367504 10.1530/REP-07-0540

[b10] Barres B. A. A role for glia in LHRH release. Curr. Biol. 2, 645–647 (1992). 15336016 10.1016/0960-9822(92)90111-m

[b11] Ojeda S. R., Lomniczi A. & Sandau U. Contribution of glial-neuronal interactions to the neuroendocrine control of female puberty. Eur. J. Neurosci. 32, 2003–2010 (2010). 21143655 10.1111/j.1460-9568.2010.07515.x PMC3058235

[b12] Sharif A., Baroncini M. & Prevot V. Role of glia in the regulation of gonadotropin-releasing hormone neuronal activity and secretion. Neuroendocrinology 98, 1–15 (2013). 23735672 10.1159/000351867

[b13] Zhou Y., Gunput R. A. & Pasterkamp R. J. Semaphorin signaling: progress made and promises ahead. Trends Biochem. Sci. 33, 161–170 (2008). 18374575 10.1016/j.tibs.2008.01.006

[b14] Pasterkamp R. J. Getting neural circuits into shape with semaphorins. Nat. Rev. Neurosci. 13, 605–618 (2012). 22895477 10.1038/nrn3302

[b15] Messina A. *et al.* Dysregulation of Semaphorin7A/beta1-integrin signaling leads to defective GnRH-1 cell migration, abnormal gonadal development and altered fertility. Hum. Mol. Genet. 20, 4759–4774 (2011). 21903667 10.1093/hmg/ddr403 PMC3221532

[b16] Parkash J. *et al.* Suppression of beta1-integrin in gonadotropin-releasing hormone cells disrupts migration and axonal extension resulting in severe reproductive alterations. J. Neurosci. 32, 16992–17002 (2012). 23175850 10.1523/JNEUROSCI.3057-12.2012 PMC5238668

[b17] Vo T. *et al.* The chemorepulsive axon guidance protein semaphorin 3A is a constituent of perineuronal nets in the adult rodent brain. Mol. Cell. Neurosci. 56, 186–200 (2013). 23665579 10.1016/j.mcn.2013.04.009

[b18] Pasterkamp R. J., Kolk S. M., Hellemons A. J. & Kolodkin A. L. Expression patterns of semaphorin7A and plexinC1 during rat neural development suggest roles in axon guidance and neuronal migration. BMC Dev. Biol. 7, 98 (2007). 17727705 10.1186/1471-213X-7-98 PMC2008261

[b19] Prevot V. *et al.* Definitive evidence for the existence of morphological plasticity in the external zone of the median eminence during the rat estrous cycle: implication of neuro-glio-endothelial interactions in gonadotropin-releasing hormone release. Neuroscience 94, 809–819 (1999). 10579572 10.1016/s0306-4522(99)00383-8

[b20] King J. C. & Letourneau R. J. Luteinizing hormone-releasing hormone terminals in the median eminence of rats undergo dramatic changes after gonadectomy, as revealed by electron microscopic image analysis. Endocrinology 134, 1340–1351 (1994). 8119174 10.1210/endo.134.3.8119174

[b21] Page R. in: Physiology of Reproduction eds Knobil E., Neill J. D. 1527–1620(Raven Press (1994).

[b22] Mullier A., Bouret S. G., Prevot V. & Dehouck B. Differential distribution of tight junction proteins suggests a role for tanycytes in blood-hypothalamus barrier regulation in the adult mouse brain. J. Comp. Neurol. 518, 943–962 (2010). 20127760 10.1002/cne.22273 PMC2892518

[b23] Peitz M., Pfannkuche K., Rajewsky K. & Edenhofer F. Ability of the hydrophobic FGF and basic TAT peptides to promote cellular uptake of recombinant Cre recombinase: a tool for efficient genetic engineering of mammalian genomes. Proc. Natl Acad. Sci. USA 99, 4489–4494 (2002). 11904364 10.1073/pnas.032068699 PMC123675

[b24] Langlet F. *et al.* Tanycytic VEGF-A boosts blood-hypothalamus barrier plasticity and access of metabolic signals to the arcuate nucleus in response to fasting. Cell Metab. 17, 607–617 (2013). 23562080 10.1016/j.cmet.2013.03.004 PMC3695242

[b25] Hokfelt T. *et al.* DARPP-32 as a marker for D-1 dopaminoceptive cells in the rat brain: prenatal development and presence in glial elements (tanycytes) in the basal hypothalamus. Adv. Exp. Med. Biol. 235, 65–82 (1988). 2976255 10.1007/978-1-4899-2723-1_6

[b26] Kang H. R., Lee C. G., Homer R. J. & Elias J. A. Semaphorin 7A plays a critical role in TGF-beta1-induced pulmonary fibrosis. J. Exp. Med. 204, 1083–1093 (2007). 17485510 10.1084/jem.20061273 PMC2118575

[b27] Bouret S., De Seranno S., Beauvillain J. C. & Prevot V. Transforming growth factor beta1 may directly influence gonadotropin-releasing hormone gene expression in the rat hypothalamus. Endocrinology 145, 1794–1801 (2004). 14670985 10.1210/en.2003-1468

[b28] Galbiati M., Magnaghi V., Martini L. & Melcangi R. C. Hypothalamic transforming growth factor beta1 and basic fibroblast growth factor mRNA expression is modified during the rat oestrous cycle. J. Neuroendocrinol. 13, 483–489 (2001). 11412334 10.1046/j.1365-2826.2001.00659.x

[b29] Balland E. *et al.* Hypothalamic tanycytes are an ERK-gated conduit for leptin into the brain. Cell Metab. 19, 293–301 (2014). 24506870 10.1016/j.cmet.2013.12.015 PMC3936883

[b30] De Seranno S. *et al.* Vascular endothelial cells promote acute plasticity in ependymoglial cells of the neuroendocrine brain. J. Neurosci. 24, 10353–10363 (2004). 15548649 10.1523/JNEUROSCI.3228-04.2004 PMC6730291

[b31] Prevot V., Dutoit S., Croix D., Tramu G. & Beauvillain J. C. Semi-quantitative ultrastructural analysis of the localization and neuropeptide content of gonadotropin releasing hormone nerve terminals in the median eminence throughout the estrous cycle of the rat. Neuroscience 84, 177–191 (1998). 9522372 10.1016/s0306-4522(97)00537-x

[b32] Prevot V. *et al.* Estradiol coupling to endothelial nitric oxide stimulates gonadotropin-releasing hormone release from rat median eminence via a membrane receptor. Endocrinology 140, 652–659 (1999). 9927290 10.1210/endo.140.2.6484

[b33] de Seranno S. *et al.* Role of estradiol in the dynamic control of tanycyte plasticity mediated by vascular endothelial cells in the median eminence. Endocrinology 151, 1760–1772 (2010). 20133455 10.1210/en.2009-0870 PMC2850227

[b34] Salvi R. *et al.* Gonadotropin-releasing hormone-expressing neurons immortalized conditionally are activated by insulin: implication of the mitogen-activated protein kinase pathway. Endocrinology 147, 816–826 (2006). 16293665 10.1210/en.2005-0728

[b35] Igaz P. *et al.* Effects of cytokines on gonadotropin-releasing hormone (GnRH) gene expression in primary hypothalamic neurons and in GnRH neurons immortalized conditionally. Endocrinology 147, 1037–1043 (2006). 16282355 10.1210/en.2005-0729

[b36] Agnew B. J., Minamide L. S. & Bamburg J. R. Reactivation of phosphorylated actin depolymerizing factor and identification of the regulatory site. J. Biol. Chem. 270, 17582–17587 (1995). 7615564 10.1074/jbc.270.29.17582

[b37] Bamburg J. R., McGough A. & Ono S. Putting a new twist on actin: ADF/cofilins modulate actin dynamics. Trends Cell Biol. 9, 364–370 (1999). 10461190 10.1016/s0962-8924(99)01619-0

[b38] Walzer T., Galibert L., Comeau M. R. & De Smedt T. Plexin C1 engagement on mouse dendritic cells by viral semaphorin A39R induces actin cytoskeleton rearrangement and inhibits integrin-mediated adhesion and chemokine-induced migration. J. Immunol. 174, 51–59 (2005). 15611227 10.4049/jimmunol.174.1.51

[b39] Scott G. A., McClelland L. A., Fricke A. F. & Fender A. Plexin C1, a receptor for semaphorin 7a, inactivates cofilin and is a potential tumor suppressor for melanoma progression. J. Invest. Dermatol. 129, 954–963 (2009). 18987670 10.1038/jid.2008.329

[b40] Puschel A. W. GTPases in semaphorin signaling. Adv. Exp. Med. Biol. 600, 12–23 (2007). 17607943 10.1007/978-0-387-70956-7_2

[b41] Wang Y. *et al.* Plexins are GTPase-activating proteins for Rap and are activated by induced dimerization. Sci. Signal. 5, ra6 (2012). 22253263 10.1126/scisignal.2002636 PMC3413289

[b42] Polakis P. & McCormick F. Interactions between p21ras proteins and their GTPase activating proteins. Cancer Surv. 12, 25–42 (1992). 1386285

[b43] Sullivan S. D. & Moenter S. M. Prenatal androgens alter GABAergic drive to gonadotropin-releasing hormone neurons: implications for a common fertility disorder. Proc. Natl Acad. Sci. USA 101, 7129–7134 (2004). 15096602 10.1073/pnas.0308058101 PMC406477

[b44] Giacobini P. & Prevot V. Semaphorins in the development, homeostasis and disease of hormone systems. Semin. Cell Dev. Biol. 24, 190–198 (2013). 23219659 10.1016/j.semcdb.2012.11.005

[b45] Giacobini P. *et al.* Brain Endothelial Cells Control Fertility through Ovarian-Steroid-Dependent Release of Semaphorin 3A. PLoS Biol. 12, e1001808 (2014). 24618750 10.1371/journal.pbio.1001808 PMC3949669

[b46] Smith M. S., Freeman M. E. & Neill J. D. The control of progesterone secretion during the estrous cycle and early pseudopregnancy in the rat: prolactin, gonadotropin and steroid levels associated with rescue of the corpus luteum of pseudopregnancy. Endocrinology 96, 219–226 (1975). 1167352 10.1210/endo-96-1-219

[b47] Dafopoulos K. *et al.* Evidence that termination of the estradiol-induced luteinizing hormone surge in women is regulated by ovarian factors. J. Clin. Endocrinol. Metab. 91, 641–645 (2006). 16332941 10.1210/jc.2005-1656

[b48] Kasa-Vubu J. Z. *et al.* Progesterone blocks the estradiol-induced gonadotropin discharge in the ewe by inhibiting the surge of gonadotropin-releasing hormone. Endocrinology 131, 208–212 (1992). 1611998 10.1210/endo.131.1.1611998

[b49] Zhao H., Tian Z., Hao J. & Chen B. Extragonadal aromatization increases with time after ovariectomy in rats. Reprod. Biol. Endocrinol. 3, 6 (2005). 15661083 10.1186/1477-7827-3-6 PMC548297

[b50] Boon W. C., Chow J. D. & Simpson E. R. The multiple roles of estrogens and the enzyme aromatase. Prog. Brain Res. 181, 209–232 (2010). 20478440 10.1016/S0079-6123(08)81012-6

[b51] Bouret S., Prevot V., Takumi T., Beauvillain J. C. & Mitchell V. Regulation by gonadal steroids of the mRNA encoding for a type I receptor for TGF-beta in the female rat hypothalamus. Neuroendocrinology 76, 1–7 (2002). 12097811 10.1159/000063678

[b52] Campbell C. S., Schwartz N. B. & Firlit M. G. The role of adrenal and ovarian steroids in the control of serum LH and FSH. Endocrinology 101, 162–172 (1977). 558875 10.1210/endo-101-1-162

[b53] Stouffer R. L. & Hennebold J. D. in: Knobil and Neill’s Physiology of Reproduction 4th edn (eds. Plant T. M., Zeleznik J. 1023–1076Elsevier (2015).

[b54] Prevot V. *et al.* Evidence that members of the TGFbeta superfamily play a role in regulation of the GnRH neuroendocrine axis: expression of a type I serine-threonine kinase receptor for TGRbeta and activin in GnRH neurones and hypothalamic areas of the female rat. J. Neuroendocrinol. 12, 665–670 (2000). 10849211 10.1046/j.1365-2826.2000.00508.x

[b55] Tamagnone L. *et al.* Plexins are a large family of receptors for transmembrane, secreted, and GPI-anchored semaphorins in vertebrates. Cell 99, 71–80 (1999). 10520995 10.1016/s0092-8674(00)80063-x

[b56] Campbell D. S. & Holt C. E. Apoptotic pathway and MAPKs differentially regulate chemotropic responses of retinal growth cones. Neuron 37, 939–952 (2003). 12670423 10.1016/s0896-6273(03)00158-2

[b57] Bechara A. *et al.* FAK-MAPK-dependent adhesion disassembly downstream of L1 contributes to semaphorin3A-induced collapse. EMBO J. 27, 1549–1562 (2008). 18464795 10.1038/emboj.2008.86 PMC2426724

[b58] Rohm B., Rahim B., Kleiber B., Hovatta I. & Puschel A. W. The semaphorin 3A receptor may directly regulate the activity of small GTPases. FEBS Lett. 486, 68–72 (2000). 11108845 10.1016/s0014-5793(00)02240-7

[b59] Hu H., Marton T. F. & Goodman C. S. Plexin B mediates axon guidance in *Drosophila* by simultaneously inhibiting active Rac and enhancing RhoA signaling. Neuron 32, 39–51 (2001). 11604137 10.1016/s0896-6273(01)00453-6

[b60] Freeman S. A. *et al.* Cofilin-mediated F-actin severing is regulated by the Rap GTPase and controls the cytoskeletal dynamics that drive lymphocyte spreading and BCR microcluster formation. J. Immunol. 187, 5887–5900 (2011). 22068232 10.4049/jimmunol.1102233

[b61] Baroncini M. *et al.* Sex steroid hormones-related structural plasticity in the human hypothalamus. NeuroImage 50, 428–433 (2010). 19969095 10.1016/j.neuroimage.2009.11.074

[b62] Kansakoski J. *et al.* Mutation screening of SEMA3A and SEMA7A in patients with congenital hypogonadotropic hypogonadism. Pediatr. Res. 75, 641–644 (2014). 24522099 10.1038/pr.2014.23

[b63] Pasterkamp R. J., Peschon J. J., Spriggs M. K. & Kolodkin A. L. Semaphorin 7A promotes axon outgrowth through integrins and MAPKs. Nature 424, 398–405 (2003). 12879062 10.1038/nature01790

