# Supplementary material for: Semaphorin7A regulates neuroglial plasticity in the adult hypothalamic median eminence
Source: Nat Commun. 2015 Feb 27;6:6385. doi: 10.1038/ncomms7385 (PMC4351556; doi:10.1038/ncomms7385)
Supplement: Supplementary Information — Supplementary Figures 1-11. [file ncomms7385-s1.pdf]

SUPPLEMENTARY FIGURES

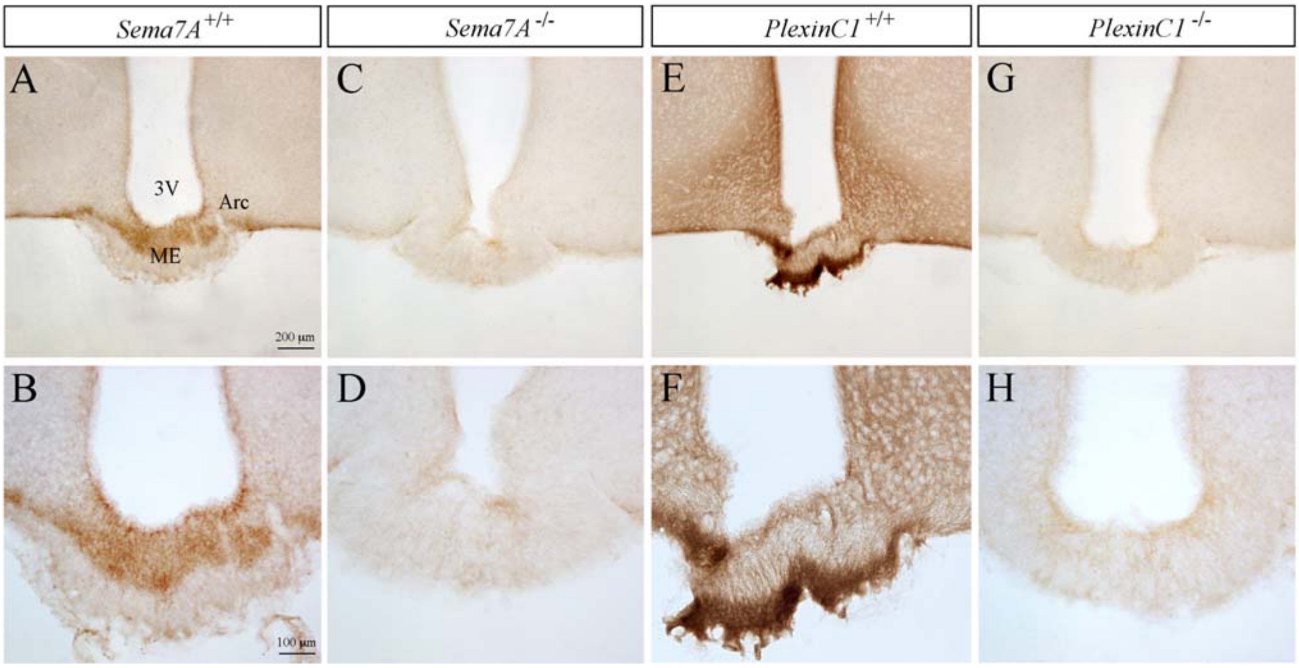

Supplementary Fig.1. Specificity of antibodies used against Sema7A and PlexinC1
(A-H) Representative coronal sections of hypothalamic median eminences of *Sema7A* (A-D, n = 3 of each genotype) and *PlexinC1* (E-H, n = 3 of each genotype) adult female (3-6 months old) deficient mice immunolabelled for Sema7A (A-D) and PlexinC1 (E-H), respectively. (A, B) Sema7A expression is robust in tanycytic cell bodies lining the third ventricle (3V) and quite diffuse along tanycytic processes spanning across the median eminence (ME) of wild-type animals. (C, D) *Sema7A*$^{-/-}$ ME is depleted of Sema7A immunoreactivity. (E, F) PlexinC1 immunoreactivity is detectable in the arcuate nucleus (Arc) of the hypothalamus and very robust in the external layer of the ME where GnRH terminals are located. (G, H) *PlexinC1*$^{-/-}$ ME is depleted of PlexinC1 immunoreactivity.

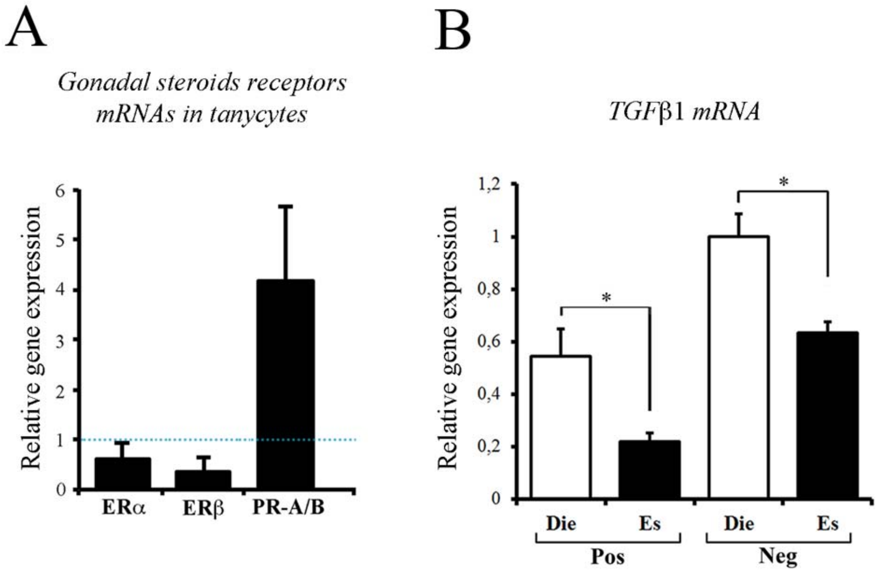

Supplementary Fig.2. Gonadal steroids receptors are expressed by adult tanycytes in female mice and TGF-β1 mRNA is upregulated in the ME at diestrus

(A, B) Tat-cre was injected into the third ventricle of adult *tdTomato*$^{loxP/+}$ female mice (4 months old). The ME was dissected and dissociated before cell sorting (FACS) of Tomato-positive cells (tanycytes) and Tomato-negative cells (astrocytes and endothelial cells) were isolated. (A) PCR analysis of estrogen receptor alpha (ERα; n = 7 *Tat-cre; tdTomato*$^{loxP/+}$), beta (ERβ; n = 7 *Tat-cre; tdTomato*$^{loxP/+}$) and progesterone receptors (PR-A/B; n = 4 *Tat-cre; tdTomato*$^{loxP/+}$) in isolated tanycytes shows that tanycytes express all three mRNAs. Values shown are means ± SEM and were normalized to Tomato-negative cells (dashed line). (B) Tomato-positive and -negative cells were isolated, respectively, at diestrus (n = 4 *Tat-cre; tdTomato*$^{loxP/+}$) and at estrus (n = 4 *Tat-cre; tdTomato*$^{loxP/+}$). Real-time PCR analysis of TGF-β1 gene was performed. Values are normalized to Tomato-negative cells at diestrus. Values shown are means ± SEM; *: $p < 0.05$, Wilcoxon-Mann-Whitney test.

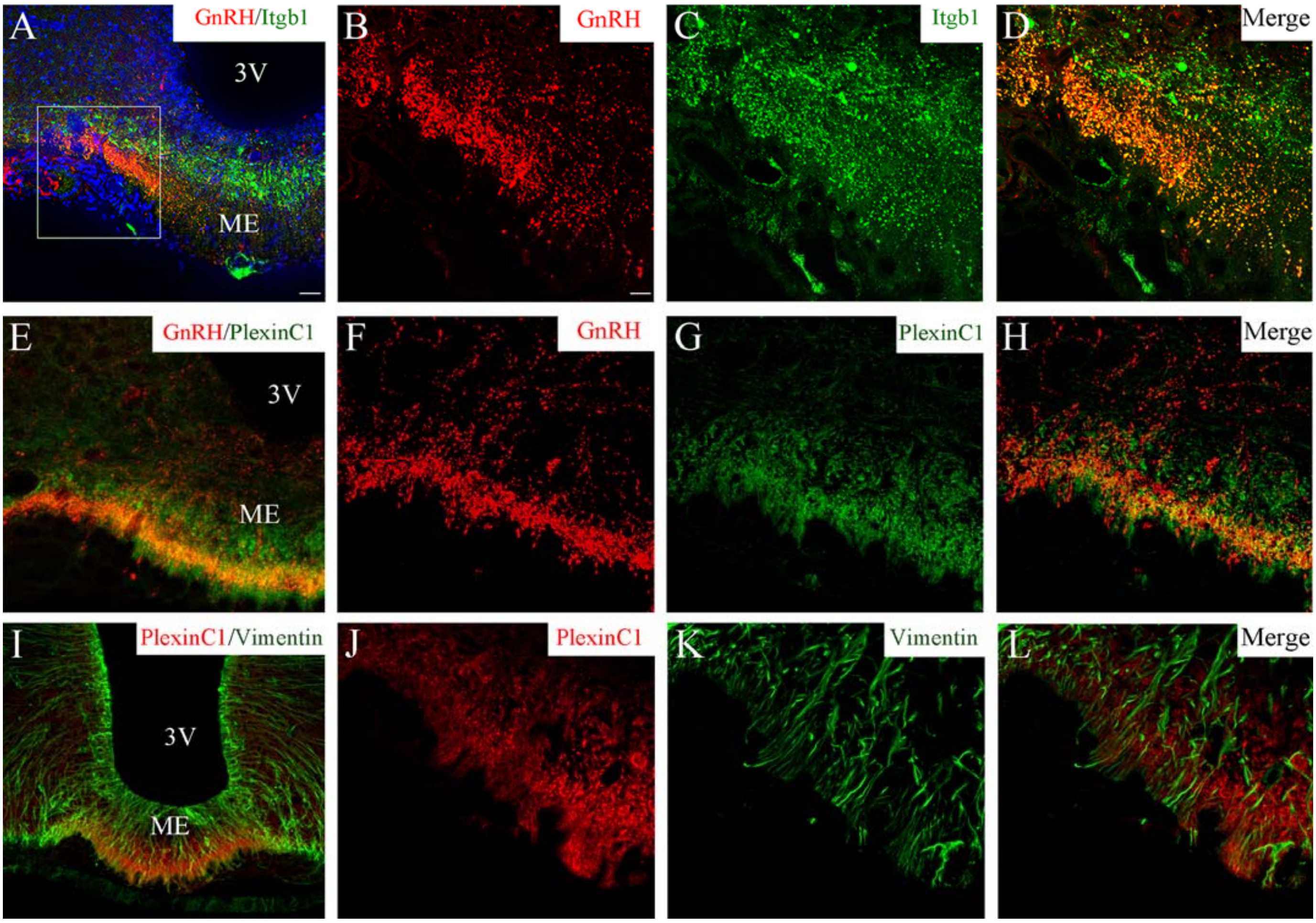

Supplementary Fig.3. Sema7A receptors are expressed in the adult hypothalamic ME
(A-H) Representative immunolabeling showing the distribution of GnRH, β1-integrin, PlexinC1 and vimentin in coronal sections of the rat adult female hypothalamic ME (n = 3, 3 months-old). (A-D) Simultaneous double-labeling experiments for GnRH (red) and β1-integrin (green) indicate colocalization of these markers in the ME. (E-H) Representative confocal images of GnRH terminals (red) and PlexinC1 (green) double-immunofluorescence. GnRH processes spanning the external zone of the ME exhibit robust PlexinC1 immunoreactivity. (I-L) Simultaneous double-labeling experiments for PlexinC1 (red) and vimentin (green) show that tanycytes (vimentin-positive) do not express PlexinC1 in the ME. 3V, third ventricle; ME, median eminence; scale bars: (A, E and I) = 50 and 100 μm respectively, (B, C, D, F, G, H, J, K, L) = 25 μm.

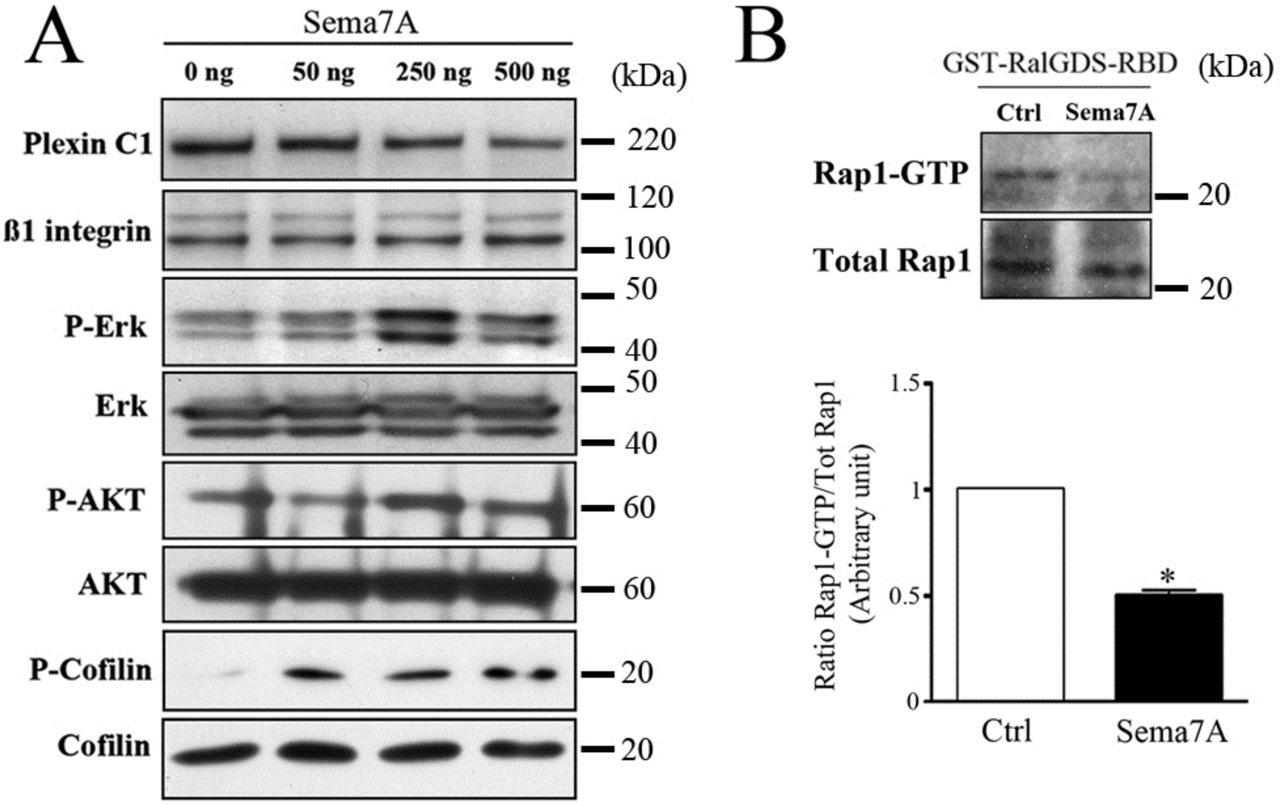

Supplementary Fig.4. Sema7A signaling pathway in GnV3 cells

(A) GnV3 cells were treated with the indicated doses of recombinant Sema7A for 30 min. Total cell lysates were resolved on 3-8% SDS–PAGE and subjected to immunoblotting with the indicated antibodies (n = 3–4 per group). (B) Pull-down assay of active Rap1 (GST-tagged Rap-Binding-Domain of RalGDS) in GnV3 cells treated or not (Ctrl) with Sema7A (250 ng/ml) for 1 minute. A representative of four independent pull-down assays with similar results is shown. Bar graphs illustrate the mean ratio of the signal intensity (optical density) obtained for Rap1-GTP to that for total Rap1 (n = 4 separate assays; Wilcoxon-Mann-Whitney test *: $p < 0.05$; values are normalized to the control groups).

Fig. 1 A Fig. 1B

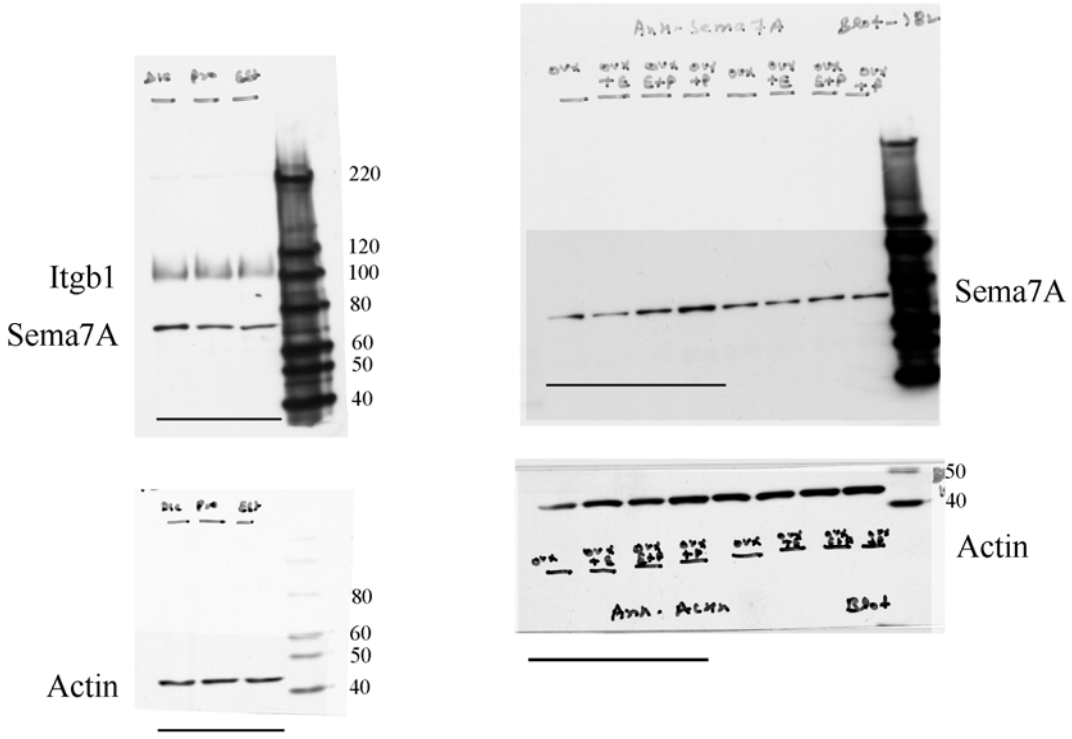

Supplementary Fig.5. Uncropped scans of the western blots presented in Figure 1.

Fig. 2A

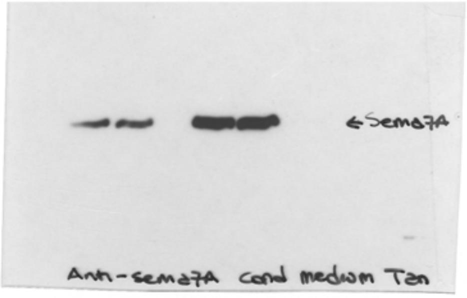

Fig. 2B

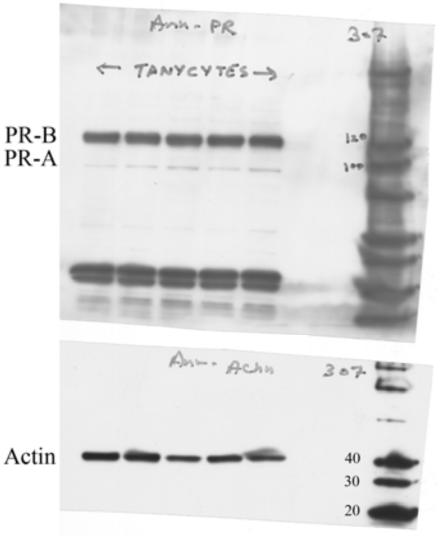

Fig. 2C

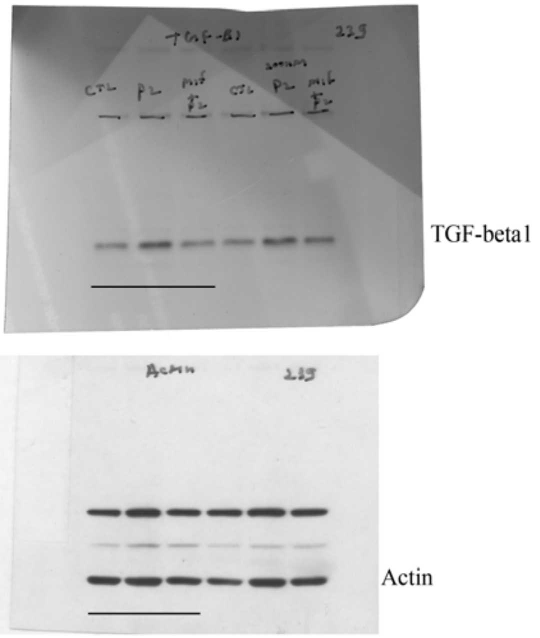

Fig. 2D

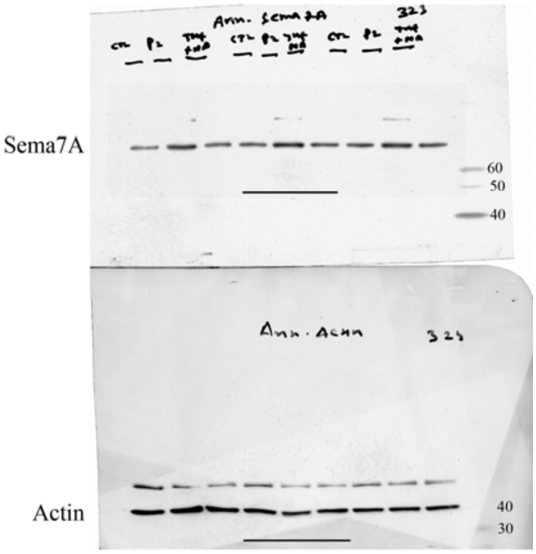

Fig. 2E

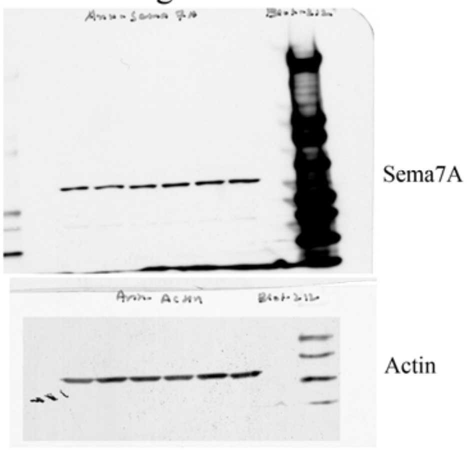

Supplementary Fig.6. Uncropped scans of the western blots presented in Figure 2.

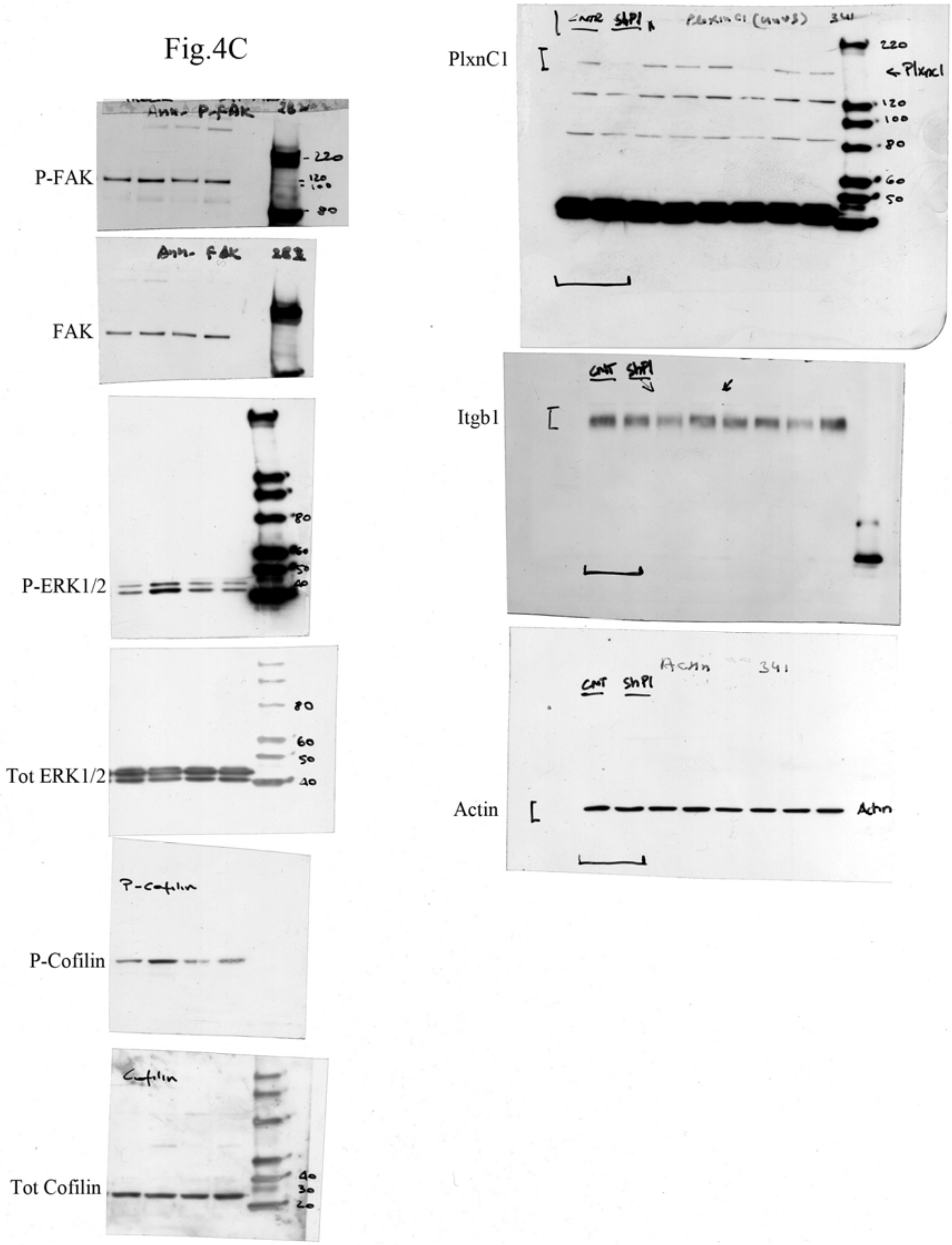

Supplementary Fig.7. Uncropped scans of the western blots presented in Figure 4.

Fig. 6A

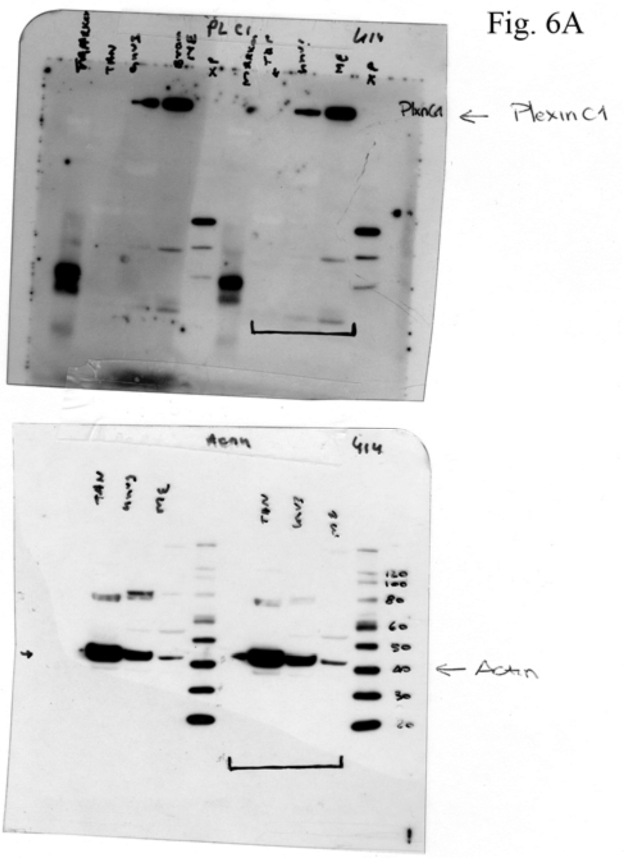

Supplementary Fig.8. Uncropped scans of the western blots presented in Figure 6A.

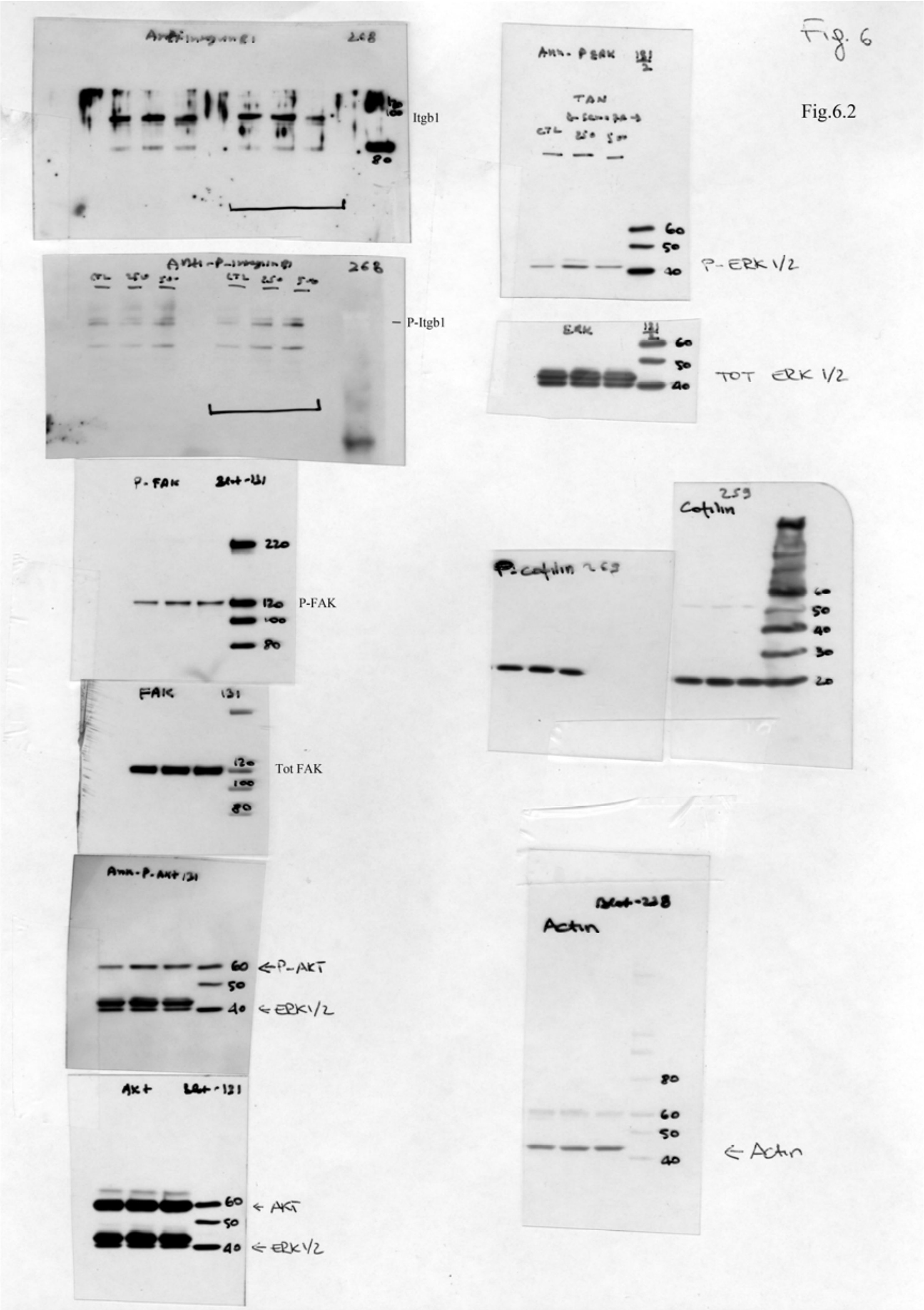

Supplementary Fig.9. Uncropped scans of the western blots presented in Figure 6B.

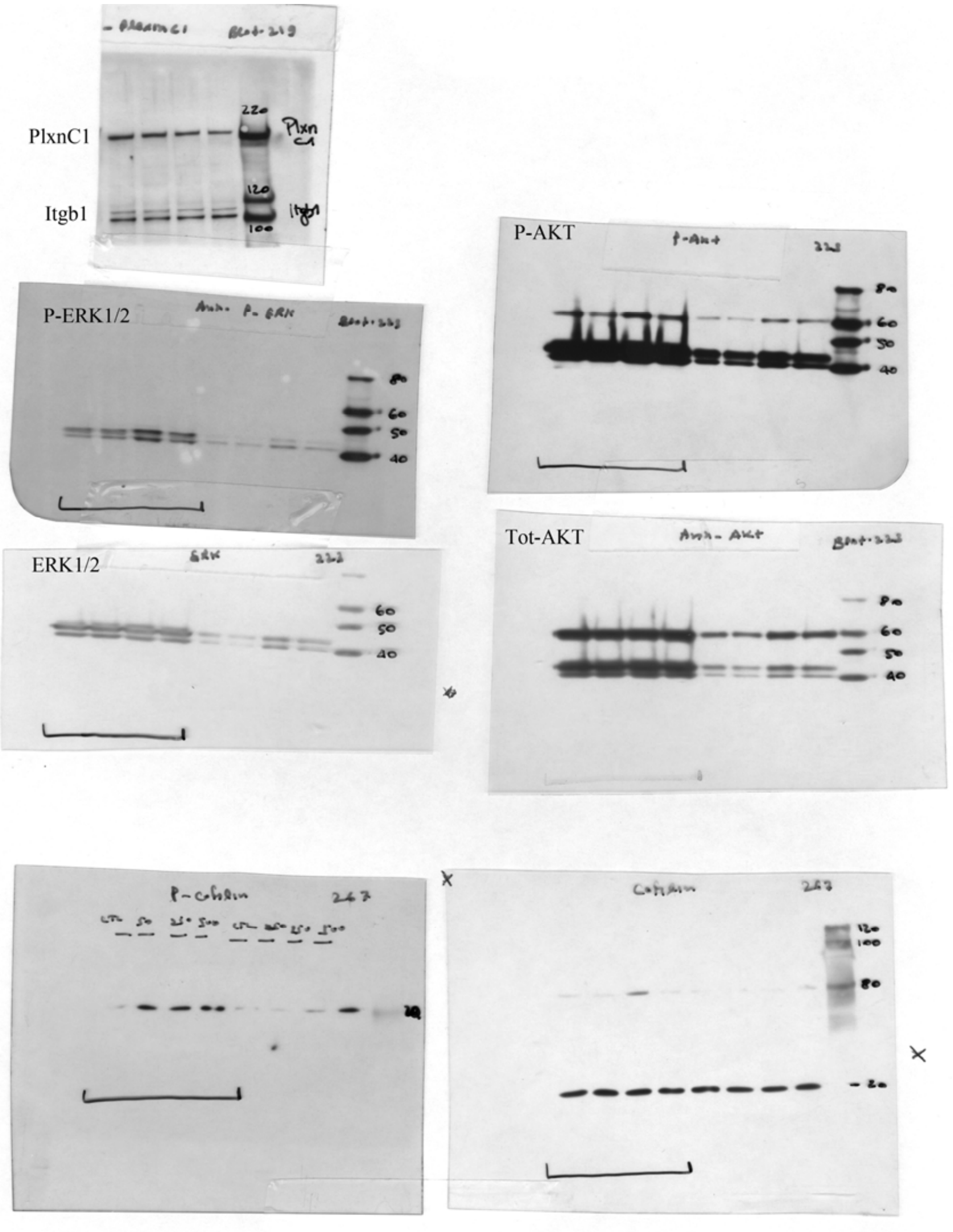

Supplementary Fig.10. Uncropped scans of the western blots presented in Figure S4A.

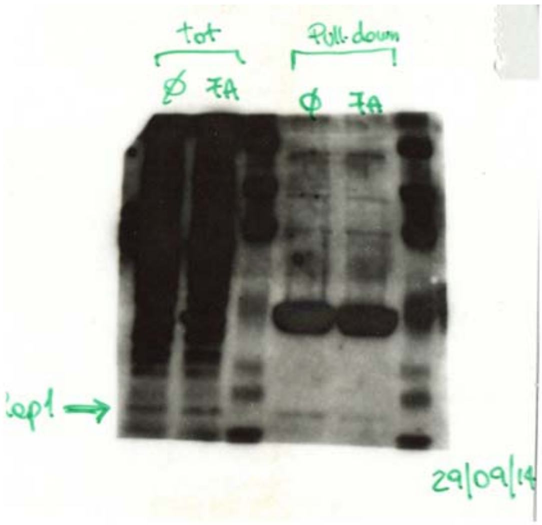

Supplementary Fig.11. Uncropped scan of the western blots presented in Figure S4B.
